# Interplay between DsbA1, DsbA2 and C8J_1298 Periplasmic Oxidoreductases of *Campylobacter jejuni* and Their Impact on Bacterial Physiology and Pathogenesis

**DOI:** 10.3390/ijms222413451

**Published:** 2021-12-15

**Authors:** Anna M. Banaś, Katarzyna M. Bocian-Ostrzycka, Stanisław Dunin-Horkawicz, Jan Ludwiczak, Piotr Wilk, Marta Orlikowska, Agnieszka Wyszyńska, Maria Dąbrowska, Maciej Plichta, Marta Spodzieja, Marta A. Polańska, Agata Malinowska, Elżbieta Katarzyna Jagusztyn-Krynicka

**Affiliations:** 1 Department of Bacterial Genetics, Faculty of Biology, Institute of Microbiology, University of Warsaw, 02-096 Warsaw, Poland; anna.banas@biol.uw.edu.pl (A.M.B.); kasia.bocian@gmail.com (K.M.B.-O.); ak.wyszynska@uw.edu.pl (A.W.); m.dabrowska@biol.uw.edu.pl (M.D.); 2 Laboratory of Structural Bioinformatics, Centre of New Technologies, University of Warsaw, 02-097 Warsaw, Poland; s.dunin-horkawicz@cent.uw.edu.pl (S.D.-H.); j.ludwiczak@cent.uw.edu.pl (J.L.); 3 Laboratory of Bioinformatics, Nencki Institute of Experimental Biology, 02-093 Warsaw, Poland; 4 Malopolska Centre of Biotechnology, Jagiellonian University, 30-387 Cracow, Poland; wilk.piotr@uj.edu.pl; 5 Faculty of Chemistry, University of Gdańsk, 80-308 Gdansk, Poland; marta.orlikowska@ug.edu.pl (M.O.); marta.spodzieja@ug.edu.pl (M.S.); 6 Laboratory of Biological Chemistry of Metal Ions, Institute of Biochemistry and Biophysics, Polish Academy of Sciences, 02-106 Warsaw, Poland; mplichta@ibb.waw.pl; 7 Department of Animal Physiology, Faculty of Biology, Institute of Functional Biology and Ecology, University of Warsaw, 02-096 Warsaw, Poland; martap@biol.uw.edu.pl; 8 Mass Spectrometry Laboratory, Institute of Biochemistry and Biophysics, Polish Academy of Sciences, 02-106 Warsaw, Poland; esme@ibb.waw.pl

**Keywords:** disulfide bond, thiol oxidoreductase, Dsb proteins, *Campylobacter jejuni*, crystal structure, substrates

## Abstract

The bacterial proteins of the Dsb family catalyze the formation of disulfide bridges between cysteine residues that stabilize protein structures and ensure their proper functioning. Here, we report the detailed analysis of the Dsb pathway of *Campylobacter jejuni*. The oxidizing Dsb system of this pathogen is unique because it consists of two monomeric DsbAs (DsbA1 and DsbA2) and one dimeric bifunctional protein (C8J_1298). Previously, we showed that DsbA1 and C8J_1298 are redundant. Here, we unraveled the interaction between the two monomeric DsbAs by in vitro and in vivo experiments and by solving their structures and found that both monomeric DsbAs are dispensable proteins. Their structures confirmed that they are homologs of EcDsbL. The slight differences seen in the surface charge of the proteins do not affect the interaction with their redox partner. Comparative proteomics showed that several respiratory proteins, as well as periplasmic transport proteins, are targets of the Dsb system. Some of these, both donors and electron acceptors, are essential elements of the *C. jejuni* respiratory process under oxygen-limiting conditions in the host intestine. The data presented provide detailed information on the function of the *C. jejuni* Dsb system, identifying it as a potential target for novel antibacterial molecules.

## 1. Introduction

The formation of disulfide bonds by the oxidation of thiol groups of cysteines is an essential step in the folding process for diverse classes of extracytoplasmic proteins, many of which are critical virulence factors. Although the process occurs spontaneously, in vivo it is catalyzed by the family of Dsb (disulfide bond) proteins located outside the cytoplasm of bacterial cells; in the periplasm or in the space between the inner membrane and the cell wall, in the case of Gram-negative and Gram-positive bacteria, respectively. The Dsb machinery is found in many various classes of microorganisms, such as Actinobacteria, Bacilli and Alpha-, Beta- and Gammaproteobacteria. In the best-characterized machinery, the *Escherichia coli* K-12 strain EcDsbA introduces disulfides into newly translocated proteins. It acts in cooperation with the inner membrane EcDsbB. Generally, DsbA generates disulfide bonds between consecutive cysteine residues. However, to achieve a proper conformation, some proteins need disulfide bonds between non-consecutive cysteine residues. Therefore, the non-native disulfides generated by EcDsbA are reshuffled by the isomerase EcDsbC, which allows correct protein folding. EcDsbC is kept in its active reduced form by EcDsbD, which transports electrons from the cytoplasm to the periplasm. Several excellent review papers present details of the process [1,2,3].

The EcDsbA structure was solved at the end of the previous century and now, two decades later, the number of solved monomeric DsbA structures from various bacterial species has grown to more than twenty. EcDsbA consists of two domains—the thioredoxin (TRX) domain and the helical domain. The catalytically active site, with a CPHC motif, is surrounded by three loops (L1, L2 and L3). The tip of L2 contains a conserved cis-proline (cis-Pro), which is part of the EcDsbA active site. The surface surrounding the active site creates a hydrophobic groove that interacts with the EcDsbA redox partner EcDsbB [4].

The unique hallmark of all characterized DsbAs, even though some display low levels of amino acid sequence identity with the prototypical EcDsbA, is the presence of the helical domain inserted into the middle of the thioredoxin fold. Solving the structures of several DsbAs, accompanied by a thorough biochemical and functional analysis, revealed two distinct classes, DsbA-I and DsbA-II, and both of these were, subsequently, divided into two subclasses [5]. The results of the subsequent phylogenetic analyses were consistent with structure-based classifications and revealed three clades [6]. The first clade corresponds to the DsbA-Ia class. These proteins exhibit a high (about 80%) identity of their amino acid sequences to EcDsbA, with the exception of *Proteus mirabilis* PmDsbA (59%) [7,8,9,10,11]. Members of this group, similar to EcDsbA, possess a long hydrophobic groove surrounding the active site. These DsbAs are rather homogenous with respect to their redox potentials and pKa values of the N-terminal and the surface-exposed cysteine of the CXXC motif [12]. Consistent with these features, DsbAs from this group complement a deficiency of EcDsbA in an EcDsbB-dependent manner, as generally determined by a motility assay.

The second clade (equivalent to the structurally defined DsbA-Ib class) consists of the DsbAs representative of Gamma- and Betaproteobacteria [13,14,15,16,17]. The sequence identities between these DsbAs and EcDsbA is low, between 20 and 30%. They display a number of structural differences relative to EcDsbA, in particular those related to the composition and conformation of the surface loops, which results in the truncation of the surface-exposed hydrophobic grooves on their catalytic sites.

The third clade (equivalent of the structural DsbA-II group) consists of DsbAs present in the proteomes of diverse taxonomic groups of microorganisms. Even though they adopt a classic EcDsbA fold, they differ from the prototypic EcDsbA, both in regard to sequence identity and architecture, as well as in their in vitro and in vivo activities [6,18,19,20,21].

DsbAs interact with two classes/groups of proteins; in oxidized states, they form disulfide bonds in their substrates, whereas in the reduced state, they are reoxidized by their redox partners—membrane-located DsbB proteins. DsbBs transfer electrons from DsbAs to the components of the respiratory chain, which assures the correct functioning of the Dsb system. Solving the structure of EcDsbA complexed with EcDsbB revealed that a short peptide (^96^PSPFATCDFM^106^) from the P2 DsbB loop plays a key role in the process [9,22,23]. The amino acid sequences of peptides from DsbBs cooperating with DsbAs belonging to class Ia are identical or almost identical to that of EcDsbB. Thus, these DsbAs complement the lack of EcDsbA in an EcDsbB-dependent manner [8,9]. The amino acid sequences of the fragments of DsbBs responsible for interactions with DsbAs belonging to class Ib or II differ from that of EcDsbB. The bacterial Dsb systems differ in terms of the number of DsbA- or DsbB-encoded proteins, and the interactions between them are more complicated than the interaction in the prototypical *E. coli* K12. Some strains of *E. coli* or *S. enterica* encode two pairs of thiol oxidoreductases, DsbA/DsbB and DsbL/DsbI, where DsbI is a homolog of DsbB that can replace classical DsbB to only a small extent [11,24,25,26]. The amino acid sequence of the fragment of the P2 loop of the EcDsbI (LFGVQGCST), determined by an in silico analysis does not resemble that of EcDsbB [12]. In contrast to the systems described above, *Neisseria meningitidis* encodes three DsbAs belonging to various classes, and all of them cooperate with one NmDsbB. In the proteome of *Pseudomonas aeruginosa,* two DsbAs (PaDsbA1 and PaDsbA2) of various substrate specificities and two DsbBs (PaDsbB1 and two PaDsbB2) are present. Both DsbBs are able to reoxidize both DsbAs, and their regions involved in this process have identical amino acid sequences (AQGMGSCKM) [14,15,16,19].

Bacteria from the *Campylobacter* genus are microaerophilic, spiral-shaped, Gram-negative microorganisms, classified to the class *Epsilonproteobacteria. Campylobacter jejuni* is a major cause of bacterial foodborne infections in many parts of the world. Patients infected by *C. jejuni* often experience severe gastroenteritis, accompanied by bloody mucous or watery diarrhea that lasts about a week. Nonetheless, the costs incurred by societies (antibiotic therapy, hospitalization, absence from work or school) are very high. *C. jejuni* is also considered to be the second most common cause of travelers’ diarrhea, lagging only behind strains of enterotoxigenic *E. coli* (ETEC). *C. jejuni* infection may also lead to other gastrointestinal diseases’ such as inflammatory bowel disease (IBD) or colorectal cancer. Additionally, human infection can manifest as extragastrointestinal diseases such as reactive arthritis and neurological illnesses, of which the most dangerous is Guillain-Barré syndrome (GBS). The number of reported confirmed cases of human campylobacteriosis in the EU was 220,682 in 2019. *C. jejuni* has many atypical virulence factors not found in other enteric bacteria. This bacterium is able to establish in many animals, but acts as a pathogen in the human population [27,28,29,30].

The *Campylobacter jejuni* Dsb system is unlike any other bacterial Dsb system described so far. The oxidative part of this network consists of two monomeric DsbAs (DsbA1 and DsbA2) related to EcDsbL, which cooperate with one DsbB. Additionally, a dimeric bifunctional Dsb protein, C8J_1298, which is related to HP0231 (dimeric *Helicobacter pylori* thiol oxidoreductase responsible for disulfide bond generation), can substitute for CjDsbA1, apart from being involved in the Dsb reductive pathway under certain conditions. In this work, to gain a more detailed understanding of the *C. jejuni* system, we further characterize CjDsbA1 in comparison with CjDsbA2.

## 2. Results

### 2.1. Biochemical and Functional Comparison of CjDsbA1 and CjDsbA2

Most of the members of the *Campylobacter jejuni* species encode two monomeric DsbAs, named CjDsbA1 and CjDsbA2, and one CjDsbB protein. In some strains, the *cjdsbA2* gene is truncated, lacking the CXXC catalytic motif, and is inactive [31]. Previously, we revealed that both monomeric CjDsbAs present in *C. jejuni* 81116 cells are structurally and phylogenetically more closely related to EcDsbL than to the main thiol oxidoreductase of *E. coli*, EcDsbA. Consistent with these data, both oxidoreductases similar to EcDsbL are inactive in the insulin reduction assay [24,32]. To gain insights into the functional details of both enzymes, we determined their redox potentials and oxidase activities. The redox potential was evaluated by equilibrating incubation using various ratios of reduced and oxidized glutathione (GSH/GSSG) as a reference. As CjDsbA2 does not contain tryptophan, the redox states of both CjDsbAs were not monitored fluorometrically, but by the AMS trapping method [33,34]. This agent binds to free thiols, which results in the increase in the molecular mass of the protein (490 Da per thiol) and enables the separation of the oxidized and the reduced forms of the Dsb proteins by non-reducing SDS-PAGE [34]. Redox potentials for both CjDsbA1 and CjDsbA2 were within the limit characteristic for monomeric DsbA oxidative activity (−60 mV for CjDsbA1 and −116 mV for CjDsbA2) (Supplementary Figure S1). It should be noted that the CjDsbA1 redox potential was the highest among the characterized DsbAs so far described; EcDsbL has a redox potential of −95 mV and *N. meningitidis* lipoprotein NmDsbA2 has a redox potential of −79 mV [12].

**Figure 1:**
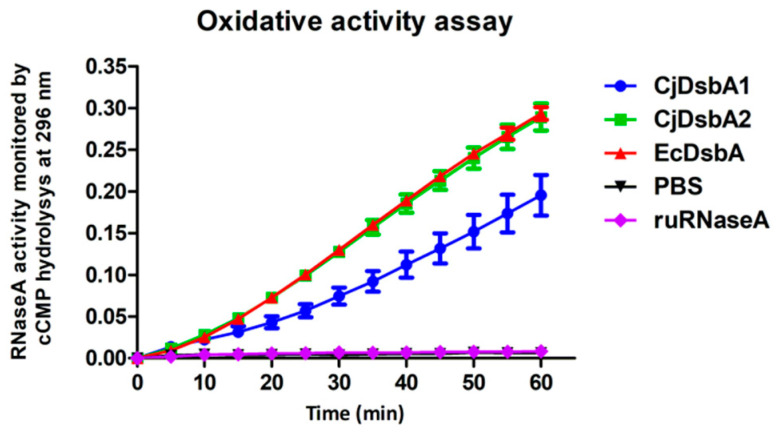
In vitro oxidase activity of CjDsbA2 was comparable to EcDsbA, but CjDsbA1 was less active in oxidative activity assay with unfolded reduced RNaseA (ruRNaseA). Purified EcDsbA was used as a control. Reactions were carried out in 200 μL of PBS buffer containing 100 mM Tris acetate pH 8.0, 2 mM EDTA, 0.2 mM GSSG, 1 mM GSH, 4.5 mM cCMP, ruRNaseA (10 μM) and the analyzed enzymes (20 μM). Changes in absorbance at 296 nm as a function of time were measured. The figure presents an average of three independent experiments, each in two technical replicates (with standard deviation error bars).

Next, we tested the ability of the CjDsbAs to introduce disulfide bonds in vitro using ribonuclease A (RNaseA) as its substrate; RNaseA needs four properly located disulfide bonds for its enzyme activity. The oxidative activity of the DsbAs was assessed by spectrophotometrically measuring the RNaseA cleavage of cCMP (cyclic-2′,3′-cytidinemonophosphate) (Figure 1) [35,36]. We found that both CjDsbAs recognized RNaseA as their substrate and exhibited oxidase activity such as that of EcDsbA, in contrast to EcDsbL that, according to literature data, does not introduce disulfides into RNaseA [24]. We observed that the CjDsbA2 activity in this test was slightly higher than that of CjDsbA1. Table 1 summarizes the biochemical features of the CjDsbAs proteins.

### 2.2. Functional Studies

**Figure 2:**
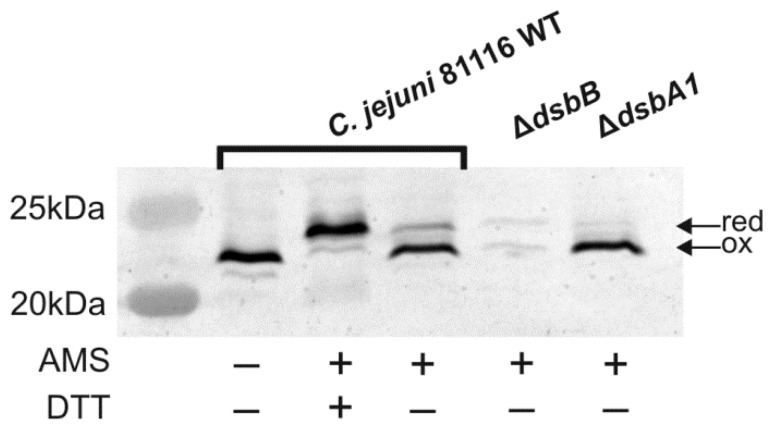
CjDsbA2 was present mainly in oxidized form in wild-type and Δ*cjdsbA1 C. jejuni* 81116 cells, whereas lack of CjDsbB resulted in almost undetectable levels of CjDsbA2. Bacterial cell cultures were treated with 10% TCA (trichloroacetic acid) and AMS (4-acetamido-4′-maleimidylstilbene-2,2′-disulfonic acid). The controls were reduced (red; treated with DTT and AMS) and oxidized (ox; untreated with AMS) wild-type cellular proteins. Samples were electrophoretically separated under non-reducing conditions using 16% polyacrylamide gels with sucrose addition. Then, Western blot analyses using specific anti-CjDsbA2 serum were performed. Three independent experiments were performed (*n* = 3). The figure presents a representative result.

The redox state of the Dsb proteins reflects their function; the Dsbs catalyzing disulfide generation is present in oxidized forms, while Dsb isomerases/reductases display reduced forms. Thus, we first compared the redox states of both CjDsbAs in their natural host—*C. jejuni* cells. Experiments were performed using the well-established AMS technique (see above) and Western blot analysis with specific polyclonal rabbit antibodies against CjDsbA1 and CjDsbA2 produced using recombinant proteins [36]. No cross-reactivity between the two antisera was observed (Supplementary Figure S2). Previously, we found that CjDsbA1 present in wild-type (wt) cells was present in its oxidized form, but it was mainly present in its reduced form in cells lacking CjDsbB; also, the lack of CjDsbA2 had no impact on its redox state ([32], see also Supplementary Figure S3). Additionally, as shown in this work, CjDsbA2 in the wt cells existed in an oxidized form. However, in contrast to CjDsbA1, which in cells lacking its redox partner, was mainly present in a reduced form, the lack of CjDsbB led to almost undetectable levels of CjDsbA2. A lack of CjDsbA1 did not affect the presence and the redox state of the CjDsbA2 (Figure 2).

**Figure 3:**
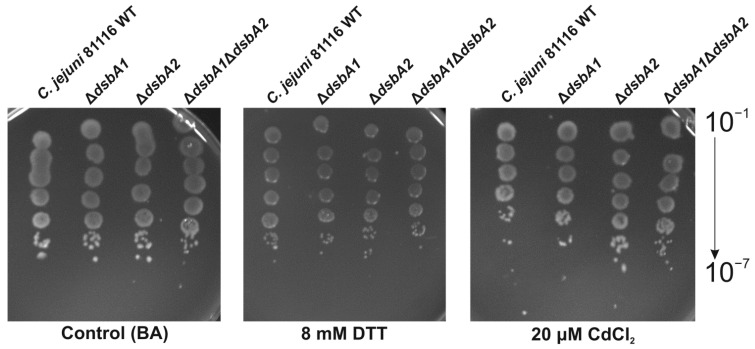
*C. jejuni* 81116 cells deficient in *cjdsbA1* or *cjdsbA2* or both *cjdsbA1* and *cjdsbA2* were not sensitive to reducing agent DTT or cadmium chloride. Serial ten-fold dilutions of *C. jejuni* 81116 strains in exponential growth phase were spotted onto BA plates supplemented with 8 mM DTT or 20 µM CdCl_2_ and incubated for 48 h at 37 °C under microaerophilic conditions. Three independent experiments were performed (*n* = 3). The figure presents a representative result.

To further explore the functional role of two CjDsbAs and to analyze the possible interplay between them, we determined the sensitivity to the reducing agent DTT of the single mutants, deficient in one or the other CjDsbAs, and a double-mutated strain lacking both CjDsbAs, using the spot-plating method (Figure 3). This assay reflects the global role of the Dsb protein in the process of disulfide generation and allows the appraisal of the ability of specific DsbA family proteins to counteract the building of incorrectly folding proteins in the periplasm. The construction of the Δ*cjdsbA1*Δ*cjdsbA2* mutant was described by Banaś et al. [36]. No enhanced DTT sensitivity compared to wt cells was seen for strains lacking CjDsbA1 or CjDsbA2 or the strain deficient in both CjDsbAs. Similarly, when we evaluated the sensitivity of strains lacking both or one DsbA to cadmium, which binds to free thiol groups, we found that the resistance of single and double mutants did not differ from the wild-type strain (Figure 3). These data indicated that both monomeric thiol oxidoreductases did not have a significant impact on the periplasmic protein redox state and suggested that another protein, probably C8J_1298, plays a major role in the process. Specific concentrations of DTT and CdCl_2_ were chosen based on previously obtained data. These concentrations clearly discriminated between wt, single (Δ*cjdsbA1,* Δ*c8j_1298*) and double (Δ*cjdsbA1*Δ*c8j_1298*)-mutated strains [36].

The phenotypic analysis was expanded by exploring the ability of the CjDsbAs to complement *E. coli* Δ*dsbA* and Δ*dsbA*Δ*dsbB* mutants. To this end, a mature *cjdsbA2* coding sequence harboring the *E. coli pelB* signal sequence was cloned into a low-copy-number vector pMPM-A6 under the control of an arabinose-inducible promoter [37]. The construct was introduced into *E. coli* Δ*dsbA* (JCB817) and into an *E. coli* strain lacking both *dsbA* and *dsbB* genes (JCB818) by transformation. The production of the CjDsbA2 protein was confirmed by Western blot analysis using polyclonal rabbit anti-CjDsbA2 antibodies (Supplementary Figure S4). The construction of *E. coli* mutants harboring CjDsbA1 was described earlier [32].

**Figure 4:**
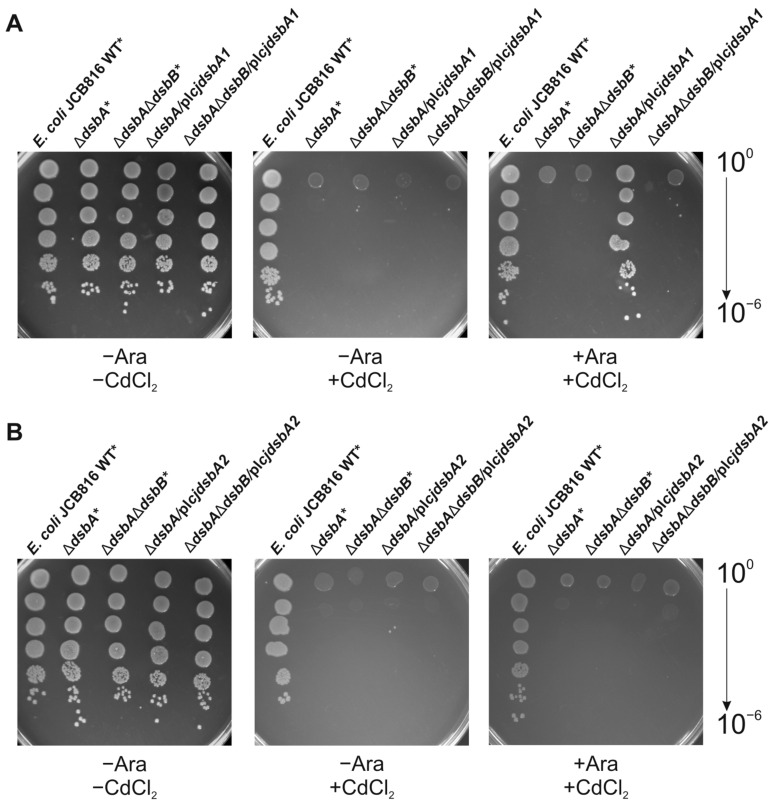
CjDsbA1 (**A**), but not CjDsbA2 (**B**), complemented lack of DsbA in DsbB-dependent manner in cadmium sensitivity assay in *E. coli* cells. *E. coli* strains: JCB816 (wild-type), JCB817 (Δ*dsbA*), JCB818 (Δ*dsbA*Δ*dsbB*), carrying pMPM-A6 plasmid (*), and Δ*dsbA*/pl*cjdsbA1*, Δ*dsbA*Δ*dsbB*/pl*cjdsbA1*, Δ*dsbA*/pl*cjdsbA2*, Δ*dsbA*Δ*dsbB*/pl*cjdsbA2* in exponential growth phase were serially diluted and spotted onto LB plates supplemented with 0.2% arabinose (+Ara) and 40 µM CdCl_2_ and incubated for 18 h at 37 °C. As controls, we used plates without arabinose (−Ara) or CdCl_2_. Three independent experiments were performed (*n* = 3). The figure presents a representative result.

**Figure 5:**
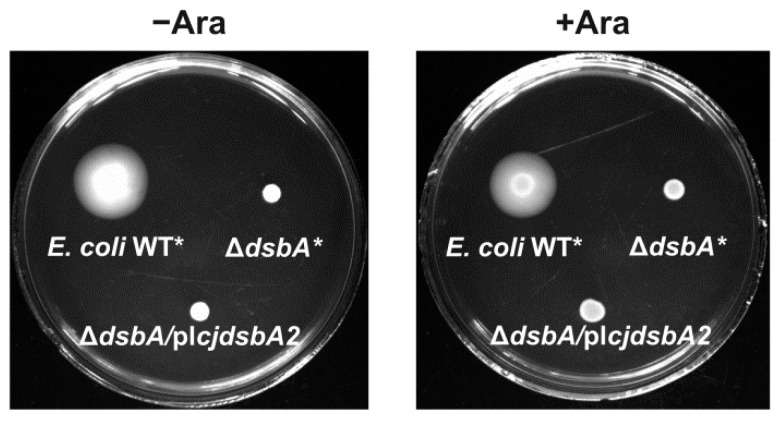
CjDsbA2 did not restore motility in *dsbA*-deficient *E. coli* strain. Exponentially growing *E. coli* JCB816 (wild-type) and JCB817 (Δ*dsbA)* both carrying pMPM-A6 plasmid (*), and Δ*dsbA*/pl*cjdsbA2* strain were spotted onto LB soft agar plates supplemented with 0.2% arabinose (+Ara) and incubated for 24 h at 37 °C. As controls, we used plates without arabinose (−Ara). Three independent experiments were performed (*n* = 3). The figure presents a representative result.

The cadmium sensitivity of the transformants was determined by the spot-plating method. We found that only CjDsbA1 restored *E. coli* Δ*dsbA* resistance to cadmium (Figure 4). Next, we checked the ability of CjDsbA2 to complement the lack of EcDsbA in a null mutant by the motility test. EcFlgL (flagellar P-ring protein), a component of the flagellum motor participating in flagella biogenesis, is a substrate of EcDsbA. As a consequence, *E. coli* Δ*dsbA* strains are non-motile [38]. In contrast to CjDsbA1 (see Grabowska et al.), CjDsbA2 did not complement the lack of EcDsbA in the motility test (Figure 5) [32].

Therefore, we decided to assess whether the monomeric DsbAs of *Campylobacter* interacted with EcDsbB.

**Figure 6:**
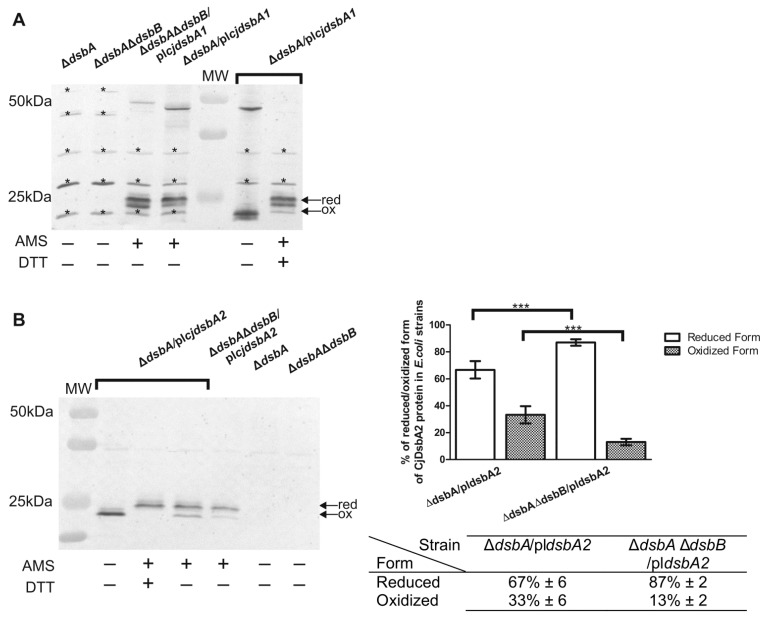
CjDsbA1 (**A**) formed dimers in *E. coli* cells. Monomeric CjDsbA1 was present in two reduced forms in both Δ*dsbA* and Δ*dsbA*Δ*dsbB,* whereas dimeric CjDsbA1 seemed to be present in oxidized form in Δ*dsbA E. coli.* CjDsbA2 (**B**) was present in both oxidized and reduced forms in *E. coli* lacking *dsbA* and mainly in reduced form *in E. coli* Δ*dsbA*Δ*dsbB*. Table and plot present quantitative analysis of equilibrium of reduced and oxidized forms of CjDsbA2 in various *E. coli* strains. Proportion of reduced and oxidized forms of CjDsbA2 was estimated using Image Lab™ (Bio-Rad) and evaluated using *t*-test (*** *p* < 0.0001) based on three independent experiments. Bacterial cell cultures were treated with 10% TCA (trichloroacetic acid) and AMS (4-acetamido-4′-maleimidylstilbene-2,2′-disulfonic acid). The controls were reduced (red; treated with DTT and AMS) and oxidized (ox; untreated with AMS) proteins. Samples were electrophoretically separated under non-reducing conditions using 16% polyacrylamide gels with sucrose addition. Then, Western blot analyses using specific anti-CjDsbA1 or anti-CjDsbA2 serum were performed. Three independent experiments were performed (*n* = 3). The figure presents a representative result. *—unspecific serum reaction; MW refers to lane with molecular weight marker.

To answer this question, the redox status of CjDsbAs in *E. coli* lacking *dsbA (E. coli* JCB817) and *E. coli* lacking both *dsbA* and *dsbB* genes *(E. coli* JCB818*)* was determined using AMS labelling. As shown in Figure 6B, CjDsbA2 in *E. coli* JCB817 was present in about 67% of the reduced and 33% of the oxidized forms, whereas CjDsbA2 in *E. coli* JCB818 accumulated mostly in the reduced form (87%), which indicated that its interaction with EcDsbB was rather weak. Thus, the lack of EcDsbA complementation may have resulted from the lack of a strong interaction between CjDsbA2 and EcDsbB. However, the lack of complementation in the motility test may have also been due to differences in the substrate specificity between CjDsbA2 and EcDsbA.

Unexpectedly, while analyzing the CjDsbA1 redox state in *E. coli* cells, we noticed significant differences compared to the CjDsbA2 redox state under identical conditions (Figure 6A). First of all, in *E. coli* JCB817, CjDsbA1 formed higher molecular weight oligomeric complexes (~46 kDa), which disappeared when cells were treated with the reducing agent DTT; in *E. coli* JCB818 lacking EcDsbB, these complexes migrated slower when compared to their mobility in *E. coli* JCB817. Oligomeric forms were not present in experiments where we evaluated the expression of CjDsbA1 in *E. coli,* since these were performed under reducing conditions (Supplementary Figure S4A). Additionally, CjDsbA1 was present in both *E. coli* strains in two reduced forms with different mobilities, possibly due to the different amount of attached AMS molecules. The reduced form of CjDsbA1 in its native host contains two cysteine residues with thiol groups, so it binds two AMS molecules. We speculated that in *E. coli,* the CjDsbA1 from the faster migrating fraction contained only one cysteine residue capable of reacting with AMS, and the second residue was presumably oxidized to sulfonic acid. The fact that oligomeric forms in *E. coli* JCB818 migrated slower compared to their mobility in *E. coli* JCB817, coupled with the observation that CjDsbA1 complemented a lack of EcDsbA in an EcDsbB-dependent manner, strongly suggested that oligomeric forms can efficiently catalyze the disulfide generation. This hypothesis was consistent with data presented by Wunderlich et al. and Ondo-Mbele et al., who showed that EcDsbA with the second cysteine residue of the CPHC motif changed to alanine, revealing activity comparable to that of the wt enzyme [39,40]. Further studies are required to determine the details of this process. However, the data presented indicate that slight differences in monomeric DsbAs structures (see below) can affect their functioning, both in native and non-native hosts. CjDsbA1 in *E. coli* was more susceptible to oxidation than CjDsbA2, and CjDsbA2 was almost undetectable in *C. jejuni* cells in the absence of CjDsbB.

### 2.3. Structure of C. jejuni DsbA Proteins

CjDsbA1 crystallized in the C2 and P2_1_2_1_2_1_ space group containing two monomers in the ASU for the monoclinic form and one molecule per ASU for the orthorhombic form, while CjDsbA2 crystallized in P3_2_21 with a single chain in the ASU. In the monoclinic CjDsbA1, the C-terminal HisTag as well as 3–4 N-terminal residues could not be modelled, and in chain B, two residues (108–109) on the surface could not be modelled. In the orthorhombic CjDsbA1, the electron density map allowed for the building of the residue range 5–193. The entire length of the polypeptide could be traced for CjDsbA2, except for the C-terminal HisTag.

The superposition of monoclinic and orthorhombic forms of CjDsbA1 showed relatively small differences between them, which was confirmed by an RMSD value of 0.45 Å.

**Figure 7:**
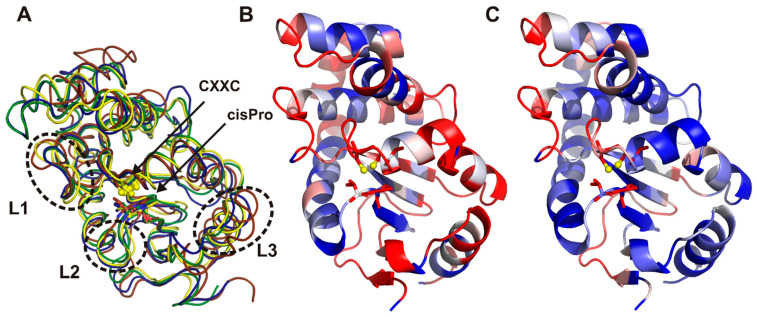
(**A**) Superposition of CjDsbA1 (yellow) and CjDsbA2 (green) compared with previously known structures of EcDsbL (blue) and EcDsbA (red). Structures are presented as tubes colored according to particular model; for consistency and clarity, the coloring was maintained throughout the manuscript. The catalytic CXXC motif and the opposing cis-proline motifs are additionally shown as sticks and colored according to element (oxygen—red; nitrogen—blue; sulfur—yellow; carbon as in the tube) and indicated by labels. Additionally, the L1, L2 and L3 regions mentioned in the text and detailed in Table 3 are indicated by dashed line ellipses and labelled accordingly. (**B**,**C**) highlight the extent of structural variability by coloring the overall structure of CjDsbA1 presented as a cartoon by per residue differences against EcDsbL (middle, panel (**B**)) and CjDsbA2 (right, panel (**C**)). Coloring based on main chain RMSD value (1.5 Å red–1.0 Å white–0.5 Å blue) between a given position in the CjDsbA1 and the compared (superposed) protein. The cis-proline motif and active site cysteines are shown as sticks with sulfur atoms presented as yellow spheres. This visually indicates higher similarity of the two *C. jejuni* proteins than similarity towards EcDsbL protein, what is also numerically expressed in Table 2.

Both *C. jejuni* DsbAs possess a thioredoxin fold with an α-helical domain inserted into the middle of the thioredoxin fold, which is recognized as a typical feature of this class of proteins. A comparison between two *E. coli* Dsbs (EcDsbA and EcDsbL) and the two *C. jejuni* Dsb proteins showed that the EcDsbA was most different, both at the primary and tertiary structure levels (Table 2). In particular, the EcDsbA L2 loop was shorter and the L3 loop was longer in comparison to both EcDsbL and CjDsbAs (Figure 7 and Table 3). These loops not only have influence on the DsbA enzymatic activities, but also are predicted to take part in binding to the periplasmic P2 loop of the partner redox protein, DsbB, and they govern interactions with the substrate proteins [8,41,42].

**Figure 8:**
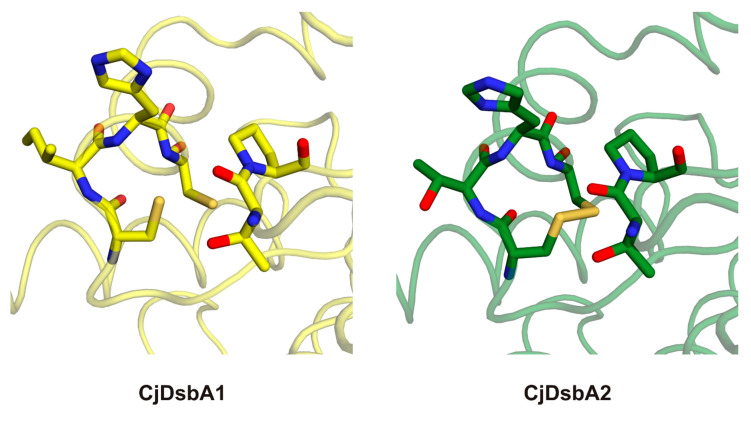

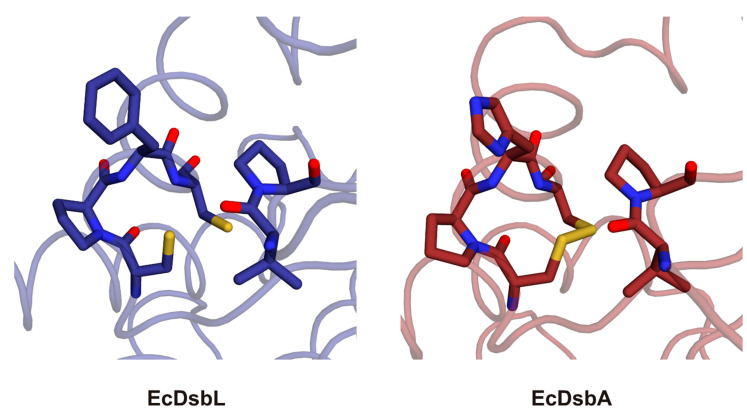
Comparison of active sites of CjDsbA1 (yellow) and CjDsbA2 (green) with previously known EcDsbL (blue) and EcDsbA (red). Main chain trace is shown as a tube and CXXC and cis-proline motifs are shown as sticks.

**Figure 9:**
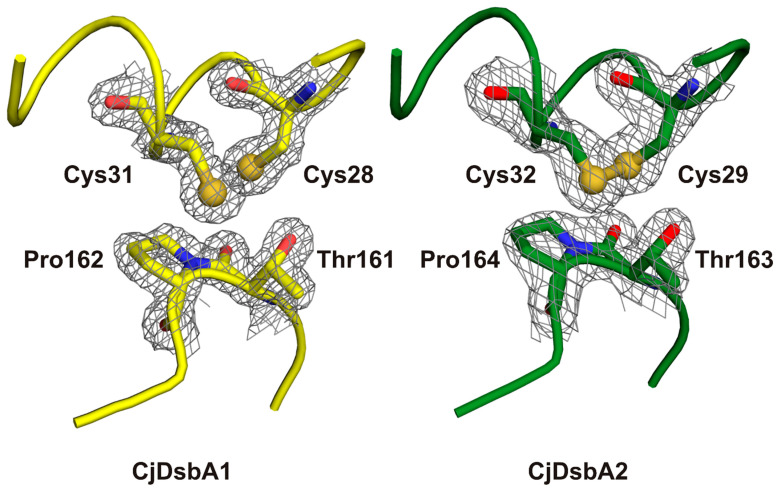
Active site of CjDsbA1 (**left**) and CjDsbA2 (**right**) exhibits different oxidation states. Selected residues are shown as sticks and 2fo–fc composite omit maps countered at 2σ level are shown as grey mesh.

The active site of DsbA proteins is highly conserved and is composed of a CXXC motif opposing a cis-proline motif. The two cysteine residues oscillates between the reduced and catalytically active oxidized form. Of note, in both *E. coli* enzymes, but not in the *C. jejuni* enzymes, the N-terminal cysteine of the CXXC motif was followed by a proline residue, which is known to play a role in the rigidification of polypeptides. Interestingly, the small hydrophobic side chain of valine preceding the cis-proline in the *E. coli* enzymes was replaced by an equally small, but charged, threonine in the *C. jejuni* enzymes (Figure 8 and Table 3). The hydroxylic group of threonine was posed in the direction of the thiol group of the acidic (more N-terminal) cysteine [43], which could possibly influence the redox potential or play a role in transition state stabilization. The active site of CjDsbA1 was observed in the reduced form, while the CjDsbA2 formed a clear disulfide bond (Figure 9). This was due to differences in protein purification procedures. CjDsbA1 was purified under reducing conditions. In contrast, the CjDsbA2 experiment was performed under native conditions (without DTT), as the CjDsbA2 reduced form did not bind to the Ni-charged resin. Additionally, the observed effect may have been caused by the prolonged process of CjDsbA2 crystallization (several months) and spontaneous air oxidation or, alternatively, by a photoreduction in CjDsbA1 during X-ray data collection. However, no other signs of radiation damage were observed.

**Figure 10:**
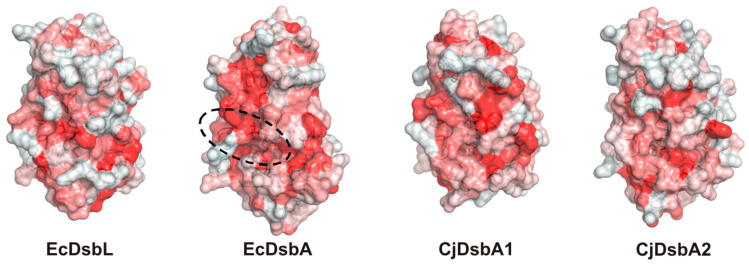
Comparison of surface hydrophobicity of selected Dsb enzymes. All structures shown in the same orientation. The EcDsbB peptide binding site is indicated by black dashed ellipse for the EcDsbA model. Red color indicates hydrophobic character.

Upon completing a catalytic reaction, the DsbA has to be reoxidized to sustain its catalytic potential. This reactivation is catalyzed by the partner, membrane-bound DsbB. As demonstrated for EcDsbA, the interaction is mediated via a hydrophobic groove on the surface of DsbA [41]. The hydrophobic character of the surface appeared to be maintained, although it seemed to be weaker for both *C. jejuni* proteins (Figure 10).

### 2.4. Cooperation of CjDsbB with CjDsbA1 and CjDsbA2

**Figure 11:**
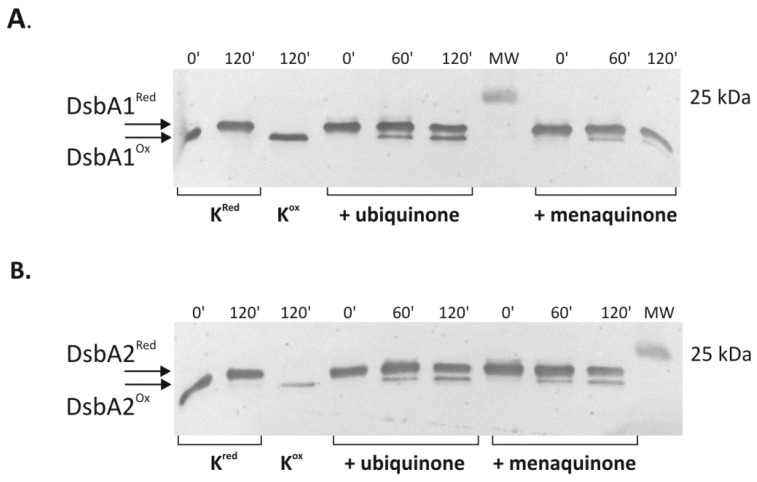
Western blot analysis of the CjDsbA1 (**A**) and CjDsbA2 (**B**) redox state in the presence of CjDsbB. The reoxidation of CjDsbA1 (**A**) or CjDsbA2 (**B**) by CjDsbB was performed in the presence of ubiquinone or menaquinone. The reaction was monitored over time by AMS trapping. Proteins were separated in 16% polyacrylamide gels with the addition of sucrose under reducing conditions and then detected by Western blot analysis using anti-CjDsbA1 antibodies (**A**) and anti-CjDsbA2 antibodies (**B**). The experimental conditions are described in Materials and Methods Section. Lanes marked ‘‘K^red^’’ and ‘‘K^ox^’’ indicate samples of reduced or oxidized DsbA1 (**A**) or DsbA2 (**B**). Lane marked MW contains molecular weight marker. Abbreviations: red—reduced; ox—oxidized.

Next, we sought to gain a more detailed understanding of the interactions between two CjDsbAs and their redox partner CjDsbB. To address this issue, we studied the ability of the detergent solubilized, partially purified CjDsbB to oxidize the reduced recombinant CjDsbA1 and CjDsbA2 in the presence of exogenous ubiquinone or menaquinone. The redox state of CjDsbAs labelled with AMS was monitored by following the shift of their electrophoretic mobility after one and two hours. A Western blot analysis was performed with specific rabbit antibodies against CjDsbA1 or CjDsbA2 (Figure 11). Prior to the enzymatic assay, the appropriate CjDsbB, CjDsbA1 and CjDsbA2 protein preparations were generated. The test included four experimental variants containing CjDsbA1 or CjDsbA2 oxidase (30 μM), CjDsbB protein (100 nM) and the electron acceptor, ubiquinone or menaquinone (MK7) (60 µM). After investigating available literature data, we found out that the DsbB protein is necessary to reoxidize DsbA, so we decided to exclude the experimental variant without the CjDsbB protein in our analysis [19]. The in vitro test indicated that CjDsbB oxidized both CjDsbA1 and CjDsbA2.The rates of in vitro reoxidation by CjDsbB for reduced forms of both CjDsbA1 and CjDsbA2 were similar, implying that small structural differences between the two CjDsbAs did not influence their in vitro affinity to CjDsbB.

We also attempted to evaluate the binding of CjDsbB to CjDsbA1 and CjDsbA2 by MST (microscale thermophoresis) technology. Solving the structure of the EcDsbA/EcDsbB complex revealed that the second periplasmic loop, (P2 region) of the EcDsbB is involved in the interaction with EcDsbA [22,23]. Based on this finding, it was determined that a short peptide (^98^PSPFATCDFM^107^) derived from this region (amino acids surrounding the cysteine residue) could be used to study EcDsbB’s interaction with various DsbAs, as well as to evaluate the effectiveness of the potential inhibitors of the Dsb system [8,41]. CjDsbB is predicted to possess a topology similar to EcDsbB, with four transmembrane helices (TM1-4) and two periplasmic loops (P1 and P2) [31]. However, the amino acid sequence surrounding the second cysteine of the P2 loop is unlike that of EcDsbB. It instead resembles that of EcDsbL [12]. Based on the amino acid sequence of the second periplasmic loop of CjDsbB, we designed and synthesized the short peptide (^110^PFAGVDGCRE^119^) and assessed its binding affinity for CjDsbA1 and CjDsbA2 using MST technology. Unfortunately, the thermophoretic signals in the studied protein/peptide systems were too weak to be quantified reliably (with signal amplitudes typically of 0.1–0.2%).

### 2.5. Identification of Dsb System Targets

To understand the physiological role of the *C. jejuni* Dsb system and its impact on the virulence, we attempted to identify its substrates. It was documented that the lack of proteins responsible for a proper disulfide generation or -SH group protection against oxidation into sulfonic acid results in protein misfolding and, finally, leads into their degradation [44]. Thus, we compared periplasmic proteomes of *C. jejuni* wt cells and that of a double-mutant strain lacking two Dsb proteins, monomeric CjDsbA1 and dimeric C8J_1298. The rationale behind using a strain deficient in these two thiol oxidoreductases was our previous observation, that they performed redundant functions [36]. This experiment was performed on *C. jejuni* 81116, but the data analysis also included the *C. jejuni* NCTC 11168 strain because it is the best characterized strain, especially in terms of respiratory protein functioning. Cell envelope extracts were prepared by the method of Hiniker and Bardwell, which restricts the quantity of the cytoplasmic proteins [45]. The experiment was performed on three biological samples for both the wt and mutated strains. Generally, 513+/−58 proteins were detected in extracts from Δ*dsbA1*Δ*c8j_1298* cells and 402+/−107 proteins were identified in wt cell extracts. In the proteomic analyses, after quantitative and statistical analyses, we identified 82 proteins whose abundance decreased in the mutated strain relative to wt cells (ratio 0.55 or less; in each case, there was an assigned q-value score). The localization of these proteins was determined by SignalP. Forty proteins were predicted to be localized out of the cytoplasm (extracellular, outer membrane, periplasmic and inner membrane proteins). The remaining were soluble cytoplasmic proteins or proteins of unknown localization (Supplementary Table S1). Among seventeen proteins whose ratio in the mutated strain relative to wt cells was 0.55 or less, with a statistically significant q-value 0.05, 12 were extracytoplasmic—based on predicted signal sequence presence (Table 4). All, excluding C8J_0558 (PEB4), contains cysteine residues in their amino acid sequences, which validated our data.

**Figure 12:**
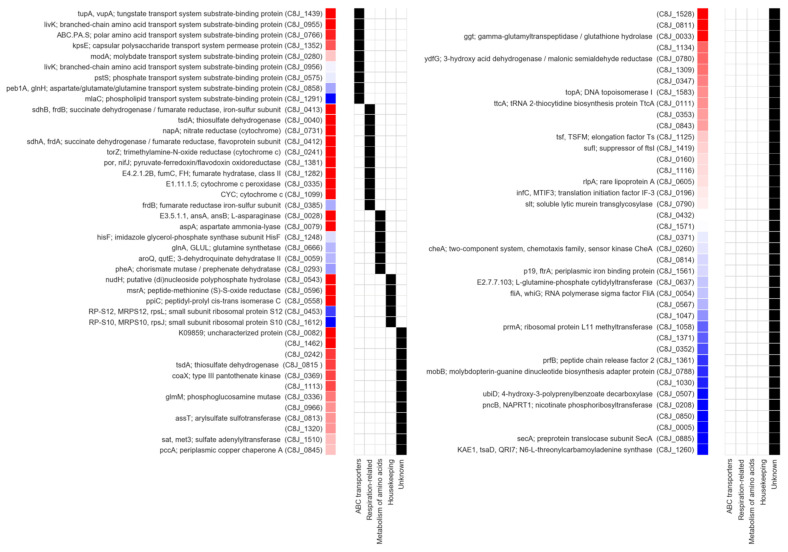
Functional annotation of 82 proteins whose ratio in the strain lacking CjDsbA1 and C8J_1298 to wild-type was 0.55 or less. For clarity, the proteins are listed in two panels. In each, the red to blue scale indicates the significance of the observed depletion (from smallest to largest, respectively), whereas the black boxes denote the functional class according to the KEGG database (the “unknown” class corresponds to the cases in which a given protein had no functional annotation in KEGG).

Next, to gain insight into the function of these proteins, we used their amino acid sequences to search the KEGG (Kyoto Encyclopedia of Genes and Genomes) database, an online resource collecting data on the function and interactions of genes and their products. As a result, 57 proteins were assigned to one of the KEGG functional groups, and 34 were further assigned to one of the KEGG pathways (Figure 12).

Based on these annotations, we identified eight periplasmic components of the ABC transporter systems. Among the most significantly depleted ones, i.e., those with the lowest q-value score, we noted TupA (C8J_1439), a periplasmic tungstate binding protein [46,47]. TupA contains two cysteine residues in positions 66 and 225 that, according to in silico modeling, may form a disulfide bridge. The proteomic analyses also revealed a drop in the abundance of two similar (>60% sequence identity) periplasmic ABC proteins, LivK (C8J_0955) and LivJ (C8J_0956), both annotated as branched amino acid transporters. For LivK, which was identified with high confidence, we performed computational modeling that indicated the presence of a disulfide bridge between residues 313 and 321. Considering the high similarity between CjLivK and CjLivJ, and the conserved position of cysteines in their sequences, one can assume that the cysteines also formed a bridge in CjLivJ (Supplementary Figure S5). The investigation of *E. coli* LivK and LivJ homologs revealed that they were also highly similar to each other (>70%) and contain conserved cysteines forming disulfide bonds, which were, however, localized differently. The universal presence of disulfide bonds in Liv proteins suggested that they may be essential for stability and explained the observed decrease in their abundance upon the disruption of the Dsb system. However, it must be noted that only some of the identified periplasmic ABC proteins contains two or more cysteines in their sequences (TupA, LivK, LivJ and C8J_0575), and the others has just one (C8J_0280 and C8J_0858) or none (C8J_1352 and C8J_1291). Out of all these, only proteins containing more than two cysteine residues were identified with statistically significant values; q-value ≤ 0.05.

We also identified several proteins related to respiratory processes, namely, two subunits (MfrA; C8J_0412 and MfrB; C8J_0413) of fumarate reductase (previously recognized as succinate dehydrogenase); nitrate reductase NapA (C8J_0731); trimethylamine-N-oxide reductase TorA (C8J_0241); cytochrome c peroxidase (C8J_0335), an enzyme with a controversial function (see discussion); two cytochromes (C8J_1099 and C8J_0040); (with low confidence) the iron–sulfur subunit of the fumarate reductase (C8J_0385). Despite functional differences, most of these proteins are either located in the periplasm (C8J_0731, C8J_0241, C8J_1099, C8J_0040 and C8J_0335) or are anchored to the inner membrane but face the periplasm (C8J_0412, C8J_0413 and C8J_0385). In contrast to the ABC transporters, all of these respiratory-related proteins contains two or more cysteines. However, structure modeling suggested that only two of them may actually contain disulfide bridges (45–226 in MfrA; C8J_0412 and 51–56, 59–71, and 154–210 in MrfB; C8J_0413). The investigation of the remaining models lacking the predicted bridges revealed that their cysteines were either spatially distant or involved in the formation of iron–sulfur clusters (see discussion for details). It is important to note that, despite their abundance in the cell, the inner membrane-anchored, cytoplasm-facing respiratory proteins were not identified during the analysis, suggesting the specificity of the employed procedure towards the identification of Dsb substrates.

Another of the identified functional groups encompassed seven proteins involved in the amino acid metabolism. Three of them, L-asparaginase (C8J_0028), aspartate ammonia-lyase (C8J_0079) and fumarate hydratase (C8J_1282), were identified with high confidence. All three high-confidence proteins identified with low q-values take part in tightly coupled reactions involving fumarate, which correlated with the reduction in the fumarate reductase amount. We also identified a group of proteins involved in the regulation and maintenance of homeostasis. Two of them, PpiC (C8J_0558), a peptidyl-prolyl cis-trans isomerase, and MsrA (C8J_0596), a methionine sulfoxide reductase, were isolated with a high statistical significance. C8J_0558 is a periplasmic chaperone with an impact on the folding of OMPs [48,49]. Although it does not possess cysteine residues in its amino acid sequence, the disturbance of homeostasis caused by the lack of Dsb proteins may have indirectly affected the amount of PpiC. MsrA contains four cysteines that, according to computational modeling, form a disulfide bridge (10–13). MsrA is a cytoplasmic enzyme that converts oxidized methionine to methionine, potentially through the sulfenic acid intermediate, which, finally, is reduced using thioredoxin as an electron donor, so it may temporarily be present in its oxidized form [50,51].

Finally, we found several proteins that, according to the KEEG database analysis, could not be assigned to any of the aforementioned functional groups but were identified with high confidence. Two were PflA (C8J_1462; paralyzed flagella), the element of the *C. jejuni* flagellar motor, and an inner membrane-anchored lipoprotein of an unknown function (C8J_0082) [52,53,54]. Modeling of the C8J_1462 and C8J_0082 revealed that both contains cysteine residues implicated in the formation of bridges (44–57, 537–565, 766–779 and 39–71, respectively). For the list of the remaining proteins associated with less significant q-values, refer to Supplementary Table S1.

### 2.6. Virulence Tests Using the Galleria mellonella Larvae Infection Model

**Figure 13:**
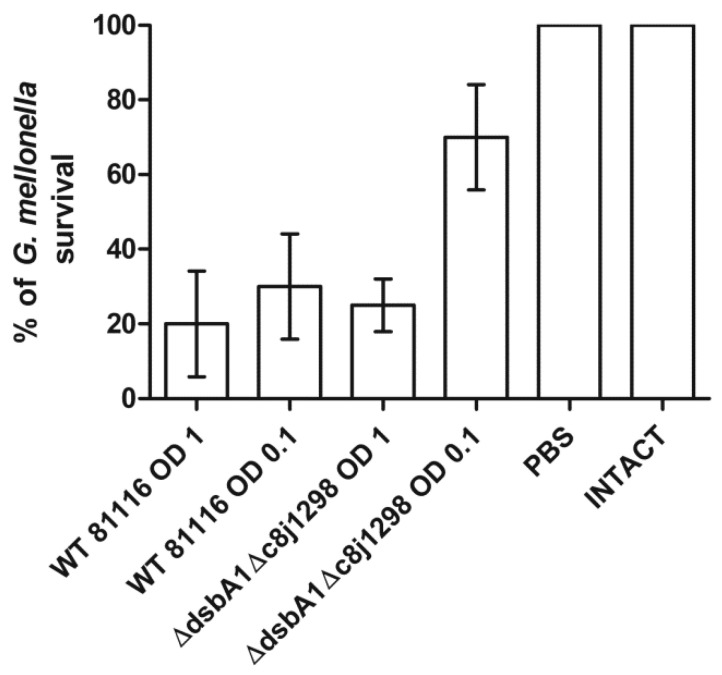
Survival of *Galleria mellonella* infected with *C. jejuni* strains. Virulence assay was performed with *C. jejuni* 81116 wild-type (WT) and double-mutated Δ*cjdsbA1*Δ*c8j_1298* strain. Larvae were injected 10^5^ (OD_590_ 0.1) or 10^6^ (OD_590_ 1) *C. jejuni*, then survival was assessed after 24 h. Larvae injected with PBS or uninjected (INTACT) were used as controls. No statistically significant differences were observed between compared groups.

Finally, we tested the global role of the disulfide bond-generating system of the *C. jejuni* in the virulence of this microorganism, using the *Galleria mellonella* insect model and measuring the ability of double-mutated strain to kill *G. mellonella*. We found there was a noticeable difference in larvae survival depending on the *C. jejuni* strain used for their inoculation (wt vs. double-mutated strain Δ*cjdsbA1*Δ*c8j_1298*). This suggested that the inactivation of the Dsb oxidative system caused a decreased *C. jejuni* virulence in the *Galleria* model. The effect was observed when *G. mellonella* larvae were infected with approximately 10^5^ bacteria (0.01 mL of the bacterial suspension in PBS of OD_590_ 0.1). Using a higher cell inoculum did not differentiate between wt and mutated bacteria (Figure 13). The level of the cytotoxicity reduction in the *C. jejuni* strain lacking two Dsb proteins to *G. mellonella* larvae was similar to that observed for mutants associated with oxidative stress described by Gundogdu et al. [55,56].

## 3. Discussion

The oxidative pathways of the Dsb systems, functioning in the cells of various bacterial genera, are extremely diverse in terms of the number of proteins, their structures and their interactions. Some bacteria have several DsbA proteins of various substrate specificities that cooperate with one or more DsbBs, while others only have a single homolog of DsbA and DsbB [1]. The biochemical, structural and phylogenetic analysis allowed the assignment of the DsbAs into three clades [6]. This division is in line with the previously proposed classification based on the biochemical features of the DsbAs proteins [5].

**Figure 14:**
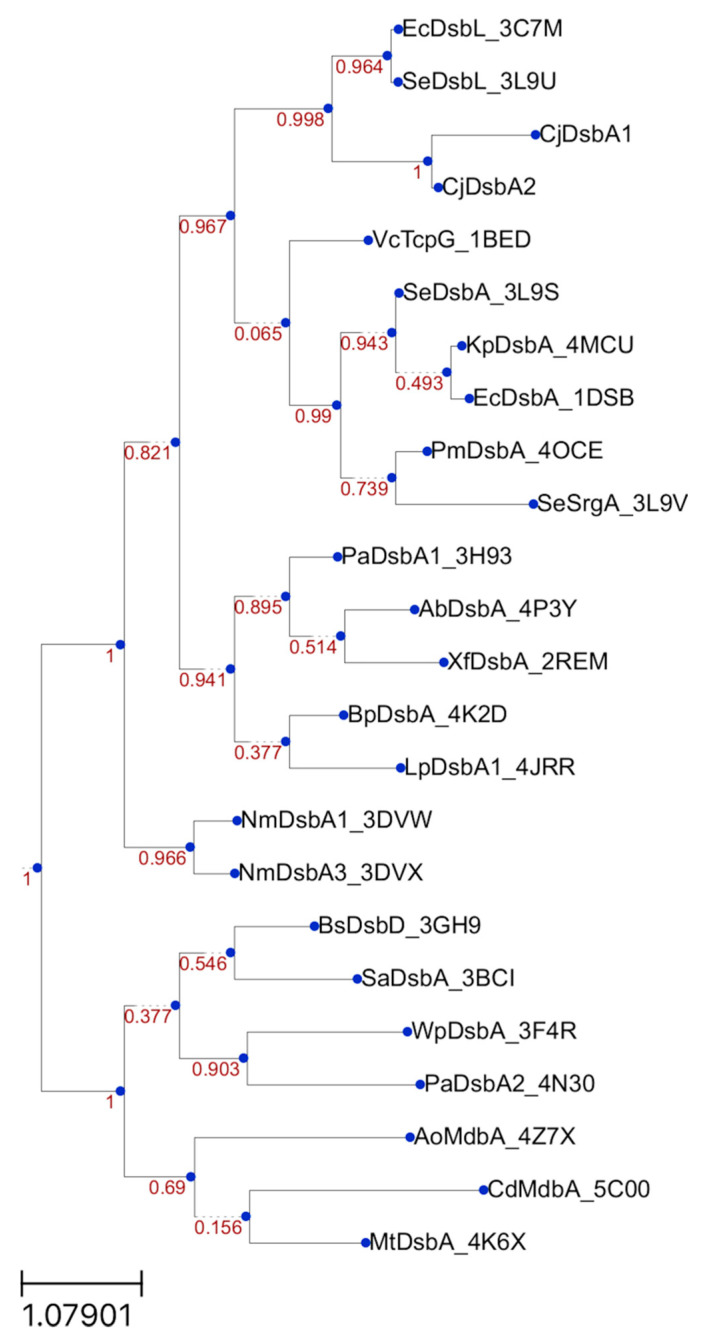
Phylogenetic tree calculated for a representative set of Dsb proteins obtained from Totsika et al. [6], and DsbA1 and DsbA1 from *C. jejuni*. Sequence names are accompanied by respective Protein Data Bank codes. Numbers near the branching points denote support values. The tree was calculated with the FastTree program and rooted using the midpoint method.

In this work, we characterized, in detail, the *C. jejuni* machinery that is responsible for maintaining the proper functional forms for cysteine residues of the periplasmic proteins. In this bacterium, there are two monomeric DsbAs, CjDsbA1 and CjDsbA2, and only one CjDsbB, encoded by a gene localized within the same transcriptional unit as *asstA* and *cjdsbA2.* Although the sequences of the active sites of CjDsbA1 and CjDsbA2 are not identical to that of EcDsbL, both of the *C. jejuni* DsbAs are considered as DsbL homologs, according to the criteria proposed by Totsika et al. (amino acid sequence identity and length of the inserted α-helical domain in the TRX fold). To define the evolutionary position of the CjDsbA proteins, we aligned their sequences with the sequences used by Totsika et al. (Figure 2 therein) and performed a phylogenetic reconstruction with the FastTree program [6,57]. In the resulting tree (Figure 14), the two CjDsbAs were localized together with EcDsbL and SeDsbL [26,32]. The solved structures of both CjDsbAs, especially the length and amino acid sequences of their L2 and L3 loops that are essential elements of the monomeric thiol oxidoreductases, were different from that of EcDsbA but similar, though not identical, to that of EcDsbL, which affected their functioning. Similar to EcDsbL, both CjDsbAs of a high redox potential were inactive in the insulin reduction test; however, in contrast to EcDsbL, but similar to EcDsbA, they were able to introduce disulfide bonds into unfolded RNaseA.

The Dsb system of *C. jejuni* is unique as it is the only known example among Gamma- and Epsilonproteobacteria where two DsbAs are closely related to the DsbL function in the absence of the classical redox pair DsbA/DsbB [32]. Our data showed that both CjDsbAs were dispensable proteins, at least under tested conditions, as a double-mutated strain lacking CjDsbA1 and CjDsbA2 did not become more sensitive to the reducing agent DTT or cadmium. Additionally, mutated strains lacking CjDsbB, in which both CjDsbAs were non-functional, did not reveal any physiological defects. In this strain, CjDsbA1 was present in the reduced forms and CjDsbA2 was almost undetectable. These results were consistent with the existence of *C. jejuni* strains possessing a non-active, truncated DsbA2 and with our previously published data that CjDsbA1 and C8J_1298 are functionally redundant [31,36]. However, we could not rule out the possibility that, under specific conditions, CjDsbA2 activity is required or beneficial. It is worth noting that, according to the in silico analysis, another species of the *Campylobacter* genus, *Campylobacter lari,* possesses a *dsbA* gene, but does not have a *dsbB* gene [32]. The mechanism of CjDsbA reoxidation remains unexplained. Considering all these data, one may suggest that the *C. jejuni dsb* genes underwent a reductive evolution process.

The conducted biochemical experiments and the similarity of the L2 and L3 loops between CjDsbA1 and CjDsbA2 indicated that both CjDsbAs possessed similar affinities to CjDsbB. Similarly, in *Neisseria meningitidis,* three DsbAs cooperate with one NmDsbB [14,58]. The CjDsbAs reoxidation reaction observed in in vitro experiments was rather slow, regardless of the quinone used (UQ or MK7), mainly because none of the quinones used in the experiment occurr naturally in the *C. jejuni* cell. Interestingly, CjDsbB revealed a higher affinity in vitro to UQ (a natural bacterial product) than to MK7 (mainly found in fermented food).

We also analyzed CjDsbAs functioning in *E. coli.* EcDsbA is the best-characterized monomeric DsbA protein. Thus, the EcDsbA complementation test that measures cell mobility is routinely used when comparing various DsbA proteins. Generally, the DsbAs of a high amino acid sequence identity to EcDsbA complement the lack of EcDsbA in this test. However, some DsbAs of a low amino acid sequence identity to EcDsbA and representing a non-EcDsbA clade are also fully or partially active in this assay [12]. We documented that CjDsbA1 complemented the lack of the EcDsbA in the motility test, in contrast to CjDsbA2, which was inactive in this assay. Additionally, the cadmium resistance test showed the same trend, i.e., CjDsbA1 complemented the lack of EcDsbA, unlike CjDsbA2, which did not. To understand differences in CjDsbA1 and CjDsbA2 functioning in *E. coli,* we checked the redox states of the proteins in *E. coli* Δ*dsbA* and *E. coli* Δ*dsbA*Δ*dsbB.* Surprisingly, despite their nearly identical structures, they reacted differently to the conditions in the *E. coli* periplasm. The extent of CjDsbA2 reoxidation by EcDsbB was rather low, and it did not complement the lack of EcDsbA in either performed test. In contrast, CjDsbA1 complemented a lack of EcDsbA in an EcDsbB-dependent manner. CjDsbA1 in *E. coli* was partially oxidized, probably due to sulfonic acid, and generated oligomeric forms (resolved by CjDsbB) that are active in disulfide generation. This suggested that CjDsbA1 uses the mechanism of creating oligomeric forms to become active in introducing disulfide bonds. This hypothesis was consistent with data presented by Wunderlich et al. and Ondo-Mbele et al., who showed that EcDsbA with the second cysteine residue of the CPHC motif changed to alanine-retained activity comparable to that of the wt enzyme [39,40]. 

A significant part of the present research was dedicated to understanding the role of the *C. jejuni* Dsb system in its physiology and virulence. To this end, we attempted to identify proteins targeted by the Dsb system by comparing the extracytoplasmic proteomes of the wt and double-mutated strains. The major constraints limiting the number of detected Dsb substrates were growth conditions and the genotype of the strains used. For example, we did not identify PhoX (alkaline phosphatase), which is a substrate of CjDsbA1, as it is produced only on minimal medium [32]. These limitations were clearly noticeable in the case of respiratory proteins. *C. jejuni* possesses extremely complex, highly branched electron transport pathways that permit the use of many different organic and inorganic electron donors, as well as a wide range of electron acceptors in addition to oxygen, which enables this microorganism to survive under a variety of conditions [59]. Two inner membrane-anchored but periplasm-facing enzymes, namely, hydrogenase (Hyd) and formate dehydrogenase (Fdh), involved in oxidation processes contain many cysteine residues. Both are key players in the *C. jejuni* metabolism in vivo as their substrates (formate and hydrogen) are produced by the intestinal microbiota and are an abundant source of electrons. The simultaneous inactivity of both Fdh and Hyd results in the severe impairment of this pathogen’s ability to colonize the chicken cecum [60,61]. However, neither was identified in our proteomic experiment, likely due to the complex media used. It was shown by van der Stel et al. that the transcription level of the *fdh* and *hyd* genes are dependent on the type of growth media and oxygen availability [62]. Formate also facilitates the microorganism’s adaptation to oxygen-limiting conditions in the host intestine [63]. Additionally, the Fdh enzyme contains tungstate, which is transported by a specific Tup system. Our data showed that the level of a periplasmic component of a Tup system, TupA protein, was clearly dependent on the Dsb oxidizing pathway, which also potentially had an impact on the Fdh level [47]. Even though these two enzymes delivering electrons to the electron transport chain were not identified in our proteomic analysis, it is highly probable that both are Dsb protein targets, since the in silico analysis revealed that their cysteine residues generated disulfide bridges. That analysis showed that FdhA had one disulfide bridge between the 60 and 92 cysteine residues, and the HydB cysteine residue 62 was connected by a disulfide bridge with the 550 cysteine.

From the proteomic experiments, we obtained a list of 82 proteins whose ratios were 0.55 or less in the mutated to wild-type strain, and 17 of them were associated with a low q-value score, indicating a high confidence. Among the 17 most certain cases, we identified mostly periplasmic components of ABC transport systems, respiratory proteins, and cytoplasmic enzymes involved in the amino acid metabolism. The functions of two additional proteins (C8J_1462 and C8J_0082) have not been precisely determined. The remaining 64 proteins had a lower confidence; however, we included them in our analyses as they represent a very similar spectrum of functions and, thus, appeared to be relevant.

Since our aim was to identify potential substrates of the Dsb system, we analyzed the top 17 proteins for the occurrence of disulfide bonds. All but one (C8J_0558—PEB4) contain at least one cysteine residue and structure modeling suggested the presence of bridges in seven of them (C8J_0082, C8J_1462, C8J_0596, C8J_0412, C8J_0413, C8J_1439 and C8J_0955).

Our data indicated two catalytic subunits (NapA and TorA) of enzymes that reduce nitrate or trimethylamine-N-oxide (TMAO), respectively, as targets of the Dsb system. Both, nitrate reductase and TMAO reductase are representative of the iron–sulfur molybdenoenzyme (CISM) family [59,64,65,66]. The NapA amino acid sequence included 11 cysteine residues. Four are part of the iron–sulfur [4Fe-4S] binding motif (C–X2–C–X3–C–X24–26–C), and Cys 176 coordinates the molybdenum center [67]. The formation of the iron–sulfur center and molybdenum binding takes place under reducing conditions of the cytoplasm, so these processes are not dependent on Dsb system activity. The role of the remaining cysteine residues and their states (oxidized vs. reduced) is still unknown. However, four of them (cysteines: 146, 166, 429, 887; the amino acid number refers to mature protein without the signal peptide) are conserved amongst NapAs from various classes of Proteobacteria, suggesting their role in protein stability or activity [64,65].

TorA is a molybdenoprotein binding the iron–sulfur (4Fe-4S) cluster necessary for electron transport. It contains eight cysteine residues occurring along the entire protein chain. In contrast to NapA, it does not possess the classical four cysteine residues involved in the iron–sulfur cluster coordination; however, some cysteine residues evidently must be involved in this process. The role of the other cysteine residues and their redox states is unknown.

Additionally, two subunits of fumarate reductase, MfrA (C8J_0412) and MfrB (C8J_0413) (previously recognized as succinate dehydrogenases), enzymes belonging to the SQOR family, were also present in significantly lesser amounts in mutated cells when compared with wt cells. Both proteins contain many cysteine residues (12 in mature MfrA and 13 in mature MfrB). However, the role of these cysteine residues in both subunits has not been the subject of research so far. Our in silico analysis showed that MfrA contained one disulfide (Cys44–Cys226—between non-consecutive cysteine residues) and, in MfrB, three disulfide bridges formed between consecutive and non-consecutive cysteine residues (Cys51–Cys56; Cys59–Cys71 and Cys154–Cys210). Clarifying the role of these disulfides requires a further analysis, though it can be assumed that the generation of disulfide bonds between non-consecutive cysteine residues depends on the isomerization activity of C8J_1298. It is interesting that our data showed a significant decrease in the amount of cytoplasmic enzymes related to the fumarate metabolism (aspartate ammonia lyase, L-asparaginase and fumarate hydratase), which correlates with the reduction in the fumarate reductase amount.

Our proteomic analysis additionally revealed three periplasmic respiratory enzymes comprising heme as a cofactor as Dsb substrates (C8J_0040, C8J_0335 and C8J_1099). Recently, it was documented that Cj0358 (a homolog of C8J_0335 in *C. jejuni* NCTC 11168) is not a detoxifying enzyme, but it can use hydrogen peroxide as an alternative to oxygen electron acceptors in respiratory processes [68]. Hydrogen peroxide seems to be an advantageous electron acceptor, especially under low-oxygen conditions that the pathogen encounters in avian or mammalian intestines, where hydrogen peroxide is released during formate oxidation and where it is produced in large amounts by commensal microflora. The next two heme-containing enzymes identified in our analysis were periplasmic c-type cytochromes, C8J_1099 (CccA) and C8J_0040, engaged in the electron transfer. *C. jejuni* encodes a large number of c-type cytochromes, among which CccA, the most abundant c-type cytochrome, plays a leading role in the process [69]. Identified proteins C8J_0040, C8J_0335 and C8J_1099 contain heme-binding motifs (CXXCH), whose cysteine residues have to be kept in a reduced state, an absolute requirement to create a covalent heme attachment. The mechanism conditioning the reduction in cysteine residues of the CXXCH under oxidizing condition of the periplasm is not completely understood. Some data indicate that C8J_1150 (a homolog of CcsX–Cj1207 in *C. jejuni* NCTC 11168) cooperating with DsbD is involved in the process, but its presence is not absolutely required [69]. Therefore, it cannot be excluded that the bifunctional protein C8J_1298, also kept in the reduced form by CjDsbD, cooperates with C8J_1150 in this process.

There were also two extracytoplasmic proteins that, in our analysis, were present only in wt cells, C8J_0082 and C8J_1462 (Cj0089 and Cj1595 in *C. jejuni* NCTC 11168). PflA encoded by *cj1559*, is a part of the *C. jejuni* flagellar motor proximal disk [54]. A mature Cj0089 is an inner membrane lipoprotein with two cysteine residues that potentially generate a disulfide bridge. The Cj0089 gene of *C. jejuni* NCTC 11168 was described by Oakland et al. as a part of the *cj0089*–*cj0090*–*cj0091* operon activated by CmeR. All proteins encoded by this operon are membrane-located lipoproteins with an undefined function involved in chicken colonization [52].

The *G. mellonella* infection model has been recently used to analyze the role of potential virulence factors in the pathogenicity of *C. jejuni* [55,70,71,72,73]. However, the interpretation of the results is difficult due to the lack of detailed data on the interaction between *C. jejuni* and *G. mellonella*. The observed effects of larvae infections depend on many factors, such as, for example, the *C. jejuni* strain or culture conditions [70,73]. Some publications provide contradictory information (survival after infection with *C. jejuni* strain 81116) [56,71,72]. In addition, some mutant strains showing attenuation in the chicken or mouse model did not increase host survival when tested in the *G. mellonella* infection model. We showed that the inactivation of the *dsb* genes impairs the virulence of *C. jejuni* as mutant strains elicit a decreased mortality of *G. mellonella* compared to wild-type strains. Several of the Dsb system substrates (respiratory or transport proteins) identified in the proteomic experiment were previously described as colonization factors of the chicken gut, making Dsb proteins possible targets for anti-microbial activity [74].

## 4. Conclusions

In this work, using a combination of biochemical, proteomic, microbiological and biophysical methods, we characterized, in detail, the *C. jejuni* Dsb system. Our data may suggest that the *C. jejuni dsb* genes underwent a reductive evolutionary process. The proteomic experiment revealed that, in addition to some virulence factors and periplasmic proteins involved in transport processes, several pivotal respiratory proteins were Dsb system targets. We also demonstrated using the *Galleria* *mellonella* insect model that the *C. jejuni* strain lacking two Dsb proteins revealed a reduced virulence. Proteins of the Dsb system, which play a key role in the virulence, potentially represent possible new drug targets. As poultry broiler meat is unquestionably the main source of human infections, new drugs that inhibit CjDsb proteins might be used as an additive in chicken food to decrease *Campylobacter* colonization in the chicken intestinal tract.

## 5. Materials and Methods

### 5.1. Bacterial Strains, Primers, Plasmids, Media and Growth Conditions

Bacterial strains, plasmids and primers used in this study are listed in Table 5. *Campylobacter jejuni* 81116 strains were grown on Blood agar base no. 2 (BA; Oxoid) plates (GenoPlast) supplemented with 5% (*v*/*v*) horse blood (ProAnimali) or on Mueller Hinton Agar (MH; Oxoid), at 37 °C under microaerobic conditions that were provided by Anoxomat Mark II OP (MART^®^ Microbiology B.V, Drachten, The Netherlands) or CampyGen (Thermo Fisher Scientific, Waltham, MA, USA). Liquid cultures were provided on BHI-SG (Brain Heart Infusion; Oxoid; supplemented with 20 mM serine and 20 mM glutamine), BHI-FBS (Brain Heart Infusion; Oxoid; supplemented with 5% (*v*/*v*) Fetal Bovine Serum; Gibco™) or Mueller Hinton Broth (MH Broth; Oxoid). All media were supplemented with Campylobacter Selective Supplement Blaser-Wang (Oxoid). For the selection of *C. jejuni* mutated and complemented strains, kanamycin (30 μg/mL) or chloramphenicol (15 μg/mL) was added to the growth media, and media were supplemented, when needed, with 20 μM CdCl_2_, 8 mM DTT. *E. coli* strains were grown at 37 °C on solid (1.5% agar), semisolid (0.35% agar) or liquid Luria-Bertani (LB) medium. When needed, media were supplemented with antibiotics at the following concentrations: 100 μg/mL ampicillin, 30 μg/mL kanamycin or/and 20 μg/mL chloramphenicol, arabinose 0.2% (*v*/*v*), glucose 1% (*v*/*v*), 40 μM CdCl_2_.

### 5.2. DNA Manipulations

#### 5.2.1. General DNA Manipulations

Standard DNA manipulations were carried out as described in the Sambrook manual or according to the manufacturer’s instructions (A&A Biotechnology, Gdańsk, Poland, New England Biolabs, Ipswich, MA, USA) [75]. Polymerase chain reactions (PCR) were performed with PrimeSTAR HS DNA Polymerase (TaKaRa) under standard conditions, according to the manufacturer’s instructions. Synthetic oligonucleotides synthesis and DNA sequencing were performed by Genomed S.A., Warsaw, Poland.

#### 5.2.2. Construction of *CjdsbA2*^+^ Plasmids for Complementation Experiments in *E. coli*

To perform complementation experiments in *E. coli* cells, *cjdsbA2* (*c8j_0811*) was cloned with *E. coli pelB* signal sequence (*ss’pelB*) under an inducible arabinose promoter. The nucleotide sequence of *cjdsbA2* (without promoter and signal sequence) was amplified from *C. jejuni* 81116 genomic DNA using a pair of primers: c8j0811Ec_BamHI–c8j0811Ec_stopXhoI. The purified PCR product and pET22b were digested with BamHI/XhoI and ligated together, forming pUWM1522. The *cjdsbA2* gene with *ss’pelB* was excised from pUWM1522 with NdeI/Klenow fragment and, subsequently, with XhoI endonuclease, then cloned into pMPM-A6 [37] digested with EcoRI/Klenow fragment and, subsequently, with XhoI endonuclease. Correctness of pUWM1523 was confirmed by sequencing and restriction analysis; then, it was transformed into *E. coli* lacking *dsbA*/ *dsbAdsbB* (JCB817/JCB818) (Table 5).

#### 5.2.3. Construction of Recombinant Plasmids for Dsb Protein Overexpression

The primers dsbB_pur_for and dsbB_pur_rev were used to amplify the 0.75 kb DNA fragment encoding CjDsbB from the chromosome of *Campylobacter jejuni* subsp. jejuni 81116 (NCTC11828—GenBank accession number CP000814) (PrimeSTAR^®^ HS DNA Polymerase (TaKaRa)). The resulting PCR product was purified and cloned into pJet1.2/blunt to generate pUWM1459 (Table 5). Thereafter, to prepare the DsbB overexpression vector, the NheI-HindIII DNA fragment of pUWM1459 and pET24a digested with NheI and HindIII was ligated. The resulting plasmid, designated pUWM1469, contained DNA-encoding CjDsbB protein with a polyhistidine tag at the C-terminus allowing single-step protein purification by Ni^2+^ affinity chromatography. Correct construction of the recombinant plasmid was verified by sequencing. Protein production was confirmed by Western blot, using anti–6His serum.

The construction of the CjDsbA1 expression vector (pUWM1430) and CjDsbA2 expression vector (pUWM1432) was described previously [36].

### 5.3. Protein Analysis and Biochemical Assays

#### 5.3.1. In Vivo Redox State

The redox states of CjDsbA1 and CjDsbA2 were determined by alkylating the free cysteine residues using 4-acetamido-4′-maleimidylstilbene-2,2′-disulfonic acid (AMS, Thermo Fisher Scientific). These agents could only modify covalently free thiols, resulting in a molecular mass increase of 0.49 kDa. The procedure was performed as previously described [34,36]. Briefly, *C. jejuni* cells were harvested from BHI-SG liquid cultures after 16–18 h of incubation under microaerobic conditions. *E. coli* cells were grown in liquid culture in LB broth until the OD_600_ value was 0.6 in standard conditions. Samples were standardized using OD_600_, then centrifuged and pellets were resuspended in sterile PBS. Next, ice-cold trichloroacetic acid (TCA, final concentration 10% *v*/*v*) was immediately added. Whole-cell proteins were precipitated and collected by centrifugation, washed twice with ice-cold acetone and then dissolved in 50 mM Tris-HCl (pH 7.5), 10 mM EDTA, 0.1% (*v*/*v*) SDS containing 10 mM AMS by agitation (1400 rpm) for 45 min at 37 °C. The proteins in non-reducing Laemmli buffer were resolved by 16% SDS-PAGE without reducing agent. Proteins were then detected by Western blot analysis using a specific serum (anti-CjDsbA1 or anti-CjDsbA2). As controls, we used samples previously treated with 100 mM DTT for 30 min at 30 °C before precipitation of the proteins with TCA.

#### 5.3.2. Overexpression and Purification

##### Overexpression and Purification of CjDsbA1, CjDsbA2 and EcDsbA

Proteins for biochemical assays and for crystallography were overexpressed in *E. coli* Rosetta (CjDsbA1 and CjDsbA2) or BL21 (EcDsbA) carrying the appropriate plasmids (Table 5) by autoinduction [80] and purified using the NGC chromatography system (Bio-Rad) as previously described [36]. Briefly, after induction, cultures were centrifuged (4000× *g*) and the cell pellet was suspended in 50 mM sodium phosphate, pH 8.0, 300 mM NaCl, 10 mM imidazole. Cells were disrupted by ultrasonication. The cell lysate was centrifuged (8000× *g*) and the resulting supernatant was applied onto Bio-Scale Mini Profinity IMAC^®^ Cartridges (Bio-Rad) containing Ni-charged resin. The protein was eluted with an imidazole gradient. To obtain a higher purity, proteins were next loaded onto ENrich^®^SEC 70 size exclusion columns (Bio-Rad) and eluted with 20 mM HEPES, 150 mM NaCl pH 7.4. For crystallography, CjDsbA1 was purified under reducing conditions (with 1 mM DTT). When needed, proteins were dialyzed on desalting columns (Bio-Rad) against PBS buffer or 20 mM HEPES, 150 mM NaCl pH 7.4 and concentrated using Amicon^®^ Ultra-4, 10,000 NMWL (Merck Millipore, Burlington, MA, USA).

##### Overexpression and Purification of CjDsbB Protein

To express CjDsbB, pUWM1469 was transformed into *E. coli* C43(DE3)-competent cells. Cells were cultured aerobically in LB medium containing 40 μg/mL of kanamycin and 1% glucose at 37 °C overnight. The next day, *E. coli* C43 cells harboring the expression vector were cultivated in fresh LB media containing kanamycin and grown at 37 °C until the OD_600_ reached about 0.5. Expression of CjDsbB was induced by adding IPTG (Thermo Scientific) to a final concentration of 250 µM, and cells were incubated at 18 °C overnight. Then, cells were harvested by centrifugation, resuspended in buffer containing 50 mM NaPi pH 8.0, 300 mM NaCl and 10% glycerol supplemented with a cocktail of protease inhibitors (Roche (complete, EDTA-free)) and disrupted by sonication (10 mL buffer/1 g pellet). The suspension obtained after centrifugation at low speed (Sorvall, 469× g, 30 min) was ultracentrifuged (Beckman, 50 Ti rotor, 110,560× *g*, 1 h, 4 °C). The pellet was homogenized in buffer containing 50 mM NaPi pH 8.0, 300 mM NaCl and 10% glycerol. N-dodecyl-β-D-maltoside (DDM) was added to the suspension of cell membranes in sonication buffer at a final concentration of 1%. After 30 min of incubation at room temperature, the membrane solution was centrifuged (15,500× *g*, 20 min) and then ultracentrifuged (Beckman, 50 Ti rotor, 110,560× *g*, 4 °C, 1 h). This supernatant was diluted twice and then incubated with a Ni-NTA His Bind^®^ Resin (Novagen) for about 3 h at 4 °C. The resin was placed in a column and washed with buffer containing 50 mM NaPi pH 8.0, 300 mM NaCl, 10% glycerol, 20 mM imidazole and 0.03% DDM. After washing the column, proteins were eluted using elution buffer (50 mM NaPi pH 8.0, 300 mM NaCl, 250 mM imidazole and 0.03% DDM). The fractions with the highest protein concentration were concentrated using a centrifugal filter (Millipore) and desalted on a Econo-Pac^®^ 10DG Desalting columns (Bio-Rad) equilibrated with buffer (50 mM NaPi pH 8.0, 300 mM NaCl and 0.1 % DDM). Finally, 1 mL of DsbB protein was obtained at a concentration of 550 µg/mL (22 µM).

#### 5.3.3. Determination the Redox Potential of CjDsbA1 and CjDsbA2 Proteins

The fractions of reduced and oxidized forms of CjDsbA1 and CjDsbA2 were determined using 4-acetamido-4′-maleimidylstilbene-2,2′-disulfonic acid (AMS) trapping [34]. Briefly, CjDsbA1 and CjDsbA2 (40 µM) were incubated overnight at room temperature in 50 mM KPi pH 7.0, 0.1 mM EDTA and various glutathione (GSH)/glutathione disulfide (GSSG) ratios. After incubation, proteins were precipitated with trichloroacetic acid (TCA, final concentration 10% (*v*/*v*)). After 20 min incubation on ice, the samples were centrifuged (16,100× *g*, 5 min, 4 °C), and the pellets were washed with cold acetone. After a second centrifugation, pellets were dried and resuspended in a buffer containing 20 mM AMS, 0.1% SDS, 10 mM EDTA and 50 mM Tris-HCl (pH 7.5). After 45 min incubation at 37 °C, with 1400 rpm shaking, samples were loaded onto 16% SDS-polyacrylamide gels under denaturing conditions. Fractions of reduced (band intensity) protein (R) were determined using Image Lab. The redox potential was calculated as described previously [36,81,82].

#### 5.3.4. Oxidative Folding of RNaseA

In vitro oxidative folding of reduced, unfolded RNaseA (ruRNaseA) was performed with CjDsbA1, CjDsbA2 and EcDsbA as described earlier [35]. Proteins were oxidized with 50 mM oxidized glutathione (GSSG) and incubated for 1 h at room temperature. RNaseA was reduced by overnight incubation at room temperature in 100 mM Tris acetate pH 8.0, containing 6 M guanidine hydrochloride and 140 mM DTT. All proteins were then dialyzed on desalting columns (Bio-Rad) and concentrated in PBS. Native RNaseA and EcDsbA were used as positive controls. Oxidase activity was measured by analyzing the cleavage of cCMP (Sigma; cytidine 2′:3′-cyclic monophosphate monosodium salt) at A296 by refolded RNaseA in the presence of tested enzymes. Reactions (triplicate) were carried out in 200 µL of PBS buffer containing 100 mM Tris acetate pH 8.0, 2 mM EDTA, 0.2 mM GSSG, 1 mM GSH, 4.5 mM cCMP, RNaseA (10 µM) and analyzed enzyme (20 µM). The reaction mixtures were prepared in a 96-well plate format and read through 30–60 min at 27 °C in a Sunrise™ (Tecan) plate reader. Three independent experiments were performed.

#### 5.3.5. Redox State Analysis of CjDsbA1 and CjDsbA2 in the Presence of CjDsbB

The ability of CjDsbB to oxidize reduced CjDsbA1 and CjDsbA2 was assayed in the presence of ubiquinone (UQ1) or menaquinone-7 (MK7). First, DsbA1 and DsbA2 were reduced with 1 mM diothiothreitol (DTT) (overnight, 4 °C). To remove DTT, the sample was applied to NAP columns (GE Healthcare) and eluted in buffer containing 50 mM NaPi pH 8.0, 300 mM NaCl and 0.1 % DDM. Reduced CjDsbA1 (30 µM) or CjDsbA2 (30 µM) was mixed with CjDsbB (100 nM) and UQ1 or MK7 (60 µM) in buffer (100 µL) containing 50 mM NaPi pH 8.0, 300 mM NaCl and 0.1% DDM. Samples (10 µL) were taken from each reaction immediately after mixing, and after 1 h and 2 h of incubation at 37 °C and precipitated with a 20% TCA solution. The supernatant was carefully discarded, pellets washed twice (200 µL 100% cold acetone) and centrifuged (20,000× *g*, 10 min). The pellets were dried at 37 °C for 20 min, suspended in buffer containing 50 mM Tris pH 7.5, 0.1% SDS, 10 mM EDTA and 10 mM AMS to label free thiols and incubated for one hour with vigorous shaking at 37 °C. The negative controls, DTT-treated CjDsbA1 or CjDsbA2 proteins, were prepared in an analogous manner. The controls for the migration of the oxidized form in the polyacrylamide gel were suspended in buffer without AMS. The proteins in non-reducing lysis buffer (312.5 mM Tris-HCl (pH = 6.8), 10% (*w*/*v*) SDS, 0.25% (*w*/*v*) bromophenol blue, 50% (*v*/*v*) glycerol) were electrophoretically separated in 16% polyacrylamide gels with the addition of sucrose. Proteins were then detected by Western blot analysis using a specific serum.

#### 5.3.6. Assessment of CjDsbA1/CjDsbA2 Interactions with CjDsbB Using Microscale Thermophoresis (MST)

CjDsbA1 and CjDsbA2 were purified as described above. Both proteins were either oxidized, by incubation with 50 mM GSSG (Sigma-Aldrich) for 30 min at 37 °C, or reduced, by incubation with 5 mM DTT (Sigma-Aldrich) for 30 min at RT, depending on the experiment. Then, the proteins were suspended in HEPES buffer (20 mM HEPES pH 7.4; 150 mM NaCl, 0.05% (*v*/*v*) TWEEN 20), using Amicon^®^ Ultra Centrifugal Filters (Millipore). Protein concentrations were determined by absorption at 280 nm, using the extinction coefficients ε_280_ = 13,040 M^−1^ cm^−1^ for CjDsbA1 and ε_280_ = 21,290 M^−1^ cm^−1^ for CjDsbA2 for the modified proteins calculated using Quest Calculate™ Protein Concentration Calculator (AAT BioQuest, Inc., Sunnyvale, CA, USA, https://www.aatbio.com/tools/calculate-protein-concentration (accessed on 30 July 2021)) [83], and later adjusted to 200 nM. Then, CjDsbA1 and CjDsbA2 were labelled with Monolith His-Tag Labeling Kit RED-tris-NTA (NanoTemper Technologies, Munich, Germany) according to the manufacturer’s manual. The CjDsbB peptide was suspended in PBS buffer with DMSO (pH 7.4; 137 mM NaCl; 2.7 mM KCl; 10 mM Na_2_HPO_4_; 1.8 mM K_2_HPO_4_; 5 mM MnCl_2_; 0.05% (*v*/*v*) TWEEN 20 and 5% (*v*/*v*) DMSO) because of its low solubility in water, to a final concentration of 1.25 mM.

To assess binding interactions between the CjDsbA1/CjDsbA2 proteins and the CjDsbB peptide, MST (microscale thermophoresis), measurements were performed using a NanoTemper ^®^ Monolith NT.115 instrument (NanoTemper Technologies). Samples were prepared according to the manufacturer’s manual and loaded in Premium coated capillaries (NanoTemper Technologies). Measurements were performed using MO.Control v1.6.1 (NanoTemper Technologies) software. Medium and high MST power conditions were applied, at 25 °C. The results were analyzed using MO.Affinity Analysis v2.3 (NanoTemper Technologies) and Origin 2019 (OriginLab Corporation, Northampton, MA, USA). Three independent measurements were performed for each sample.

### 5.4. Peptide Synthesis and Purification

The peptide was synthesized on a semiautomated peptide synthesizer (Millipore 9050 Plus PepSynthesizer, Millipore Corporation, Burlington, VT, USA) using the method of solid phase peptide synthesis (SPPS). Synthesis was performed on a TentaGel R RAM resin (0.19 mmol/g), using 9-fluorenylmethoxycarbonyl/tert-butyl (Fmoc/tBu) chemistry with the following side-chain-protected amino acid derivatives: Fmoc-Pro-OH, Fmoc-Phe-OH, Fmoc-Ala-OH, Fmoc-Gly-OH, Fmoc-Val-OH, Fmoc-Asp(OtBu)-OH, Fmoc-Cys(Trt)-OH, Fmoc-Arg(Pbf)-OH and Fmoc-Glu(OtBu)-OH. Acetylation of the N-terminal amino group of the peptide was performed using 1-acetylimidazole (1.10 g/1 g of resin) at room temperature for 24 h. Synthesized peptide was cleaved from the resin using a mixture: 88% trifluoroacetic acid (TFA), 5% H_2_O, 5% phenol and 2% triisopropylsilane (20 mL/1 g of resin) at room temperature for 3 h. After filtration of the exhausted resin, the solution was concentrated under vacuum, and the residue was triturated with Et_2_O. The precipitated peptide was centrifuged for 15 min at 2608× *g*, followed by decantation of the ether phase from the crude peptide (this process was repeated thrice). The peptide was then dissolved in H_2_O and lyophilized.

Prior to the purification process the peptide was dissolved in H_2_O and a 10-fold excess of dithiothreitol (DTT) was added. The mixture was sonified for 30 min in 60 °C. Purification of the crude peptide was carried out by using reversed phase-high performance liquid chromatography (RP-HPLC) on a semi-preparative Phenomenex Luna C8(2) (250 mm × 20 mm, 5 µm) column. A linear gradient from 5% B to 50% B in A in 180 min was applied. The aqueous system (A) consisted of 0.1% (*v*/*v*) TFA solution in water, whereas the organic phase (B) was 80% acetonitrile in water, containing 0.08% (*v*/*v*) TFA. Purification was monitored by UV absorption at a wavelength of 222 and 254 nm. The purity of the peptide was verified by LC-ESI-IT-TOF/MS (Shimadzu, Shim-pol, Warsaw, Poland), and by using RP-HPLC with a Kromasil C8 analytical column (250 mm × 4.6 mm, 5 µm), where a gradient of 5 to 100% B in A in 60 min was employed (A and B as described above).

### 5.5. Crystallization, Data Collection and Structure Determination

Protein crystals were obtained by the hanging (CjDsbA1) or sitting (CjDsbA2) drop vapor diffusion methods.

For CjDsbA1, protein solution at a concentration of 48 mg/mL was used in all crystallization experiments, and 1µL CjDsbA1 was mixed with an equal volume of crystallization buffer. Crystals were obtained in monoclinic and orthorhombic forms. Monoclinic crystals were formed in optimized Morpheus 2–33 conditions (0.08 M Carboxylic acids, 0.1 M Tris/BICINE pH 8.5, 50% (*v*/*v*) Precipitant Mix 1), while crystals of orthorhombic form were grown in Morpheus 2–9 conditions (0.12 M ethylene glycols, 0.1 M Tris/BICINE pH 8.5, 50% (*v*/*v*) Precipitant Mix 1). No cryoprotection was necessary due to the presence of cryoprotectant in the crystallization buffer. In total, 0.25 µL CjDsbA2 130 mg/mL was mixed with an equal volume of crystallization buffer. Crystals formed in LMB F5 condition (29% (*w*/*v*) PEG 4000, 0.1 M sodium citrate, 0.1 M magnesium acetate tetrahydrate, 0.1 M ammonium sulfate, pH 6.5) were flash-frozen in LN2 using mother liquor supplemented with 25% glycerol as the cryo-solution. Diffraction data were collected at crystallographic beamlines at BESSY Berlin, Elettra Sincrotrone and Swiss Light Source, respectively, and processed with XDS as implemented in XDSAPP package [84,85]. The phase problem was solved by molecular replacement. Phenix MRage pipeline was used for phasing CjDsbA1 [86,87]. The best solution, based on a model of *Salmonella enterica* DsbL (PDB ID 3L9U), was automatically rebuilt with AutoBuild [11,88]. The CjDsbA1 model was, subsequently, used as a search model in Phaser to solve the structure of CjDsbA2 [89]. All structures were manually curated in COOT and refined with phenix.refine or Refmac [90,91,92]. Relevant data collection and refinement statistics are summarized in Table 6. Images were rendered with PyMOL (Molecular Graphics System, Schrödinger, LLC, New York, NY, USA).

### 5.6. Dsb Substrate Identification—Comparison of Periplasmic C. jejuni 81116 Strains Subproteomes

#### 5.6.1. Preparation of Periplasmic Protein Extracts from *C. jejuni* 81116

*C. jejuni* cells were grown in liquid BHI-FBS (FBS 5%, *v*/*v*) at 37 °C for 16–18 h under microaerobic conditions. Periplasmic protein extracts from *C. jejuni* were prepared according to Hiniker and Bardwell with minor modifications [45]. The cell suspensions were centrifuged (5000× *g*, 20 min, 4 °C) and resulting pellets were resuspended in 1 mL of TSE buffer (0.2 M Tris-HCl pH 8.0, 0.5 M sucrose and 1 mM EDTA). Cells were incubated on ice for 30 min, transferred into Eppendorf tubes and centrifuged (16,000× *g*, 30 min, 4 °C). Resulting supernatants containing periplasmic proteins were moved into a new tube. Periplasmic proteins were then precipitated by adding trichloroacetic acid (TCA) to a final concentration 15% (*v*/*v*), incubated on ice for 30 min and centrifuged (16,000× *g*, 30 min, 4 °C). Pellets were washed with ice-cold acetone twice and dried. The samples were stored at −20 °C.

#### 5.6.2. Mass Spectrometry

Mass spectrometry was performed at the Mass Spectrometry Laboratory at the Institute of Biochemistry and Biophysics PAS. Protein pellets were subjected to a standard procedure of trypsin digestion, during which proteins were reduced with 5 mM TCEP in 100 mM ammonium bicarbonate for 1 h at 60 °C, blocked with 10 mM MMTS for 10 min at RT and digested overnight with 10 µL of 0.1 µg/µL trypsin (Promega). Resulting peptide mixtures were subjected to peptide solid-phase extraction with Waters Oasis Sample Extraction system and resuspended in 2% ACN/0.1% TFA. Peptide concentration was measured using Pierce Quantitative Colorimetric Peptide Assay (Thermo Fisher Scientific, Waltham, MA, USA ). A total of 3 µg of each sample was analyzed using LC–MS system composed of an UPLC chromatograph (nanoAcquity, Waters) directly coupled to a QExactive mass spectrometer (Thermo Fisher Scientific, Waltham, MA, USA). Peptides were trapped on C18 pre-column (180 µm × 20 mm, Waters) using 0.1% FA in water as a mobile phase and then transferred to a nanoAcquity BEH C18 column (75 µm × 250 mm, 1.7 µm, Waters) using ACN gradient (0–35% ACN in 160 min) in the presence of 0.1% FA at a flow rate of 250 nL/min. Blank runs were performed between samples to ensure lack of cross-contamination. To increase the number of peptides and proteins for analysis, separate series of measurements from pooled samples in 3 *m/z* ranges (300–600, 580–800, 780–2000) were performed. The mass spectrometry proteomics data were deposited on the ProteomeXchange Consortium via the PRIDE partner repository with the dataset identifier PXD029340 and 10.6019/PXD029340.

#### 5.6.3. Analysis of Mass Spectrometry Data

The acquired MS/MS data were preprocessed with Mascot Distiller software (v. 2.7, Matrix Science, London, UK), and a search was performed with the Mascot Search Engine (Matrix Science, London, UK, Mascot Server 2.7). The amino acid sequence database was composed of two *Campylobacter jejuni* proteomes (strains 81–176 and NCTC 11168, 3371 sequences; 1,034,205 residues) derived from UniProt and Contaminant database (115 sequences; 38,188 residues). To reduce mass errors, the peptide and fragment mass tolerance settings were established separately for individual LC-MS/MS runs after a measured mass recalibration, as described previously [93]—typical tolerance value for parent ions was 5 ppm and for daughter ions—0.01 Da. The rest of search parameters were as follows: enzyme, Trypsin; missed cleavages, 1; fixed modifications, Methylthio (C); variable modifications, Oxidation (M); instrument, HCD. A statistical assessment of the confidence of peptide assignments was based on the target/decoy database search strategy [94]. Proteins with less than two peptides and proteins identified by a subset of peptides from another protein were removed from analysis. Proteins that exactly matched the same set of peptides were combined into a single group (family). The mass calibration and data filtering described above were carried out with MScan software, developed in-house (http://proteom.ibb.waw.pl/mscan/ (accessed on 12 January 2021) [95]).

#### 5.6.4. Quantification

The lists of identified peptides were merged into one common list. This list was overlayed onto 2D heatmaps generated from LC–MS/MS datasets by tagging the peptide-related isotopic envelopes with corresponding peptide sequence tags on the basis of the measured/theoretical mass difference, the deviation from the predicted elution time and the match between theoretical and observed isotopic envelopes. A more detailed description of the quantitative extraction procedure implemented by our in-house software is available in [96]. The abundance of each peptide was determined as the height of a 2D fit to the monoisotopic peak of the tagged isotopic envelope. Quantitative values were next exported into text files, along with peptide/protein identifications, for statistical analysis with Diffprot [93] software. Diffprot was run with the following parameters: number of random peptide sets = 10 6; clustering of peptide sets—only when 90% identical; normalization by LOWESS.

### 5.7. Galleria mellonella Virulence Model

*Galleria mellonella* (Department of Animal Physiology, Faculty of Biology) at final larvae stage was maintained at 15 °C in dark. *C. jejuni* strains were incubated on Muller-Hinton broth under microaerophilic conditions for 24 h with shaking at 37 °C. Cells were harvested, washed twice in PBS and adjusted to OD_590_ 1 and ten-fold diluted to OD_590_ 0.1. Larvae were microinjected with 10 µL of *C. jejuni* inocula (using insulin syringe 30 G, BD™ Becton, Dickinson and Company) through the left hindmost proleg (infection dose was 10^6^ or 10^5^ CFU, respectively). Control experiments were performed with PBS-injected and non-injected larvae. The larvae were incubated at 37 °C and monitored at 24 h intervals [70,97].

### 5.8. Phenotype Assays

#### 5.8.1. Motility Assay

Motility assays were performed as described earlier [32]. *E. coli* cells were grown in liquid culture with shaking (150 rpm) in LB broth supplemented with 0.2% (*v*/*v*) arabinose and appropriate antibiotics until the OD_600_ value was 0.6. Then, 4 µL of bacterial culture was spotted onto LB soft agar plates containing 0.35% (*w*/*v*) agar and incubated overnight at 37 °C. All motility assays were performed in triplicate.

#### 5.8.2. Spot Titers for Cadmium and DTT Resistance

Spot titers for cadmium and DTT resistance were performed to quantify the relative oxidase activity of *C. jejuni* mutated strains*,* as previously described [36,98]. Briefly, *C. jejuni* cells were harvested from BA plates after 16–18 h of incubation under microaerobic conditions. Samples were standardized using OD_600_ of the culture and serially 10-fold diluted with LB medium. Then, 3 µL of each dilution was plated onto BA plates, supplemented with 20 µM cadmium chloride (CdCl_2_) or 8 mM DTT. *C. jejuni* cells were incubated 48 h under microaerobic conditions at 37 °C. *E. coli* cells were grown in liquid culture with shaking (150 rpm) in LB broth supplemented with 0.2% (*v*/*v*) arabinose and appropriate antibiotics until the OD_600_ value was 0.6. Then, samples were serially 10-fold diluted and 3 µL of each dilution was spotted onto LB soft agar plates containing 0.2% (*v*/*v*) arabinose and 40 µM cadmium chloride and incubated overnight at 37 °C. The DTT agar plates were used within 40 min after preparation. All spot titers were performed in triplicate.

### 5.9. Bioinformatic Analysis

Mass Spectrometry data were assigned to UniProt protein’s IDs for *C. jejuni* NCTC 11168 or *C. jejuni* 81-176. To obtain IDs for *C. jejuni* 81116 proteins BLAST search were performed [99].

Presence of the signal peptides in the analyzed proteins was assessed with the SignalP version 5.0 and further confirmed with newly released SignalP 6.0 [100,101]. 

Structural modelling of the sequences was performed with the RoseTTAFold protocol [102]. The obtained ensembles of 5 models per each of the analyzed sequence were then visually inspected in PyMOL for occurrence of disulfide bridges in the regions that the algorithm marked as high-confidence predictions.

### 5.10. Statistical Analysis

Statistical analyses were performed using GraphPad Prism (GraphPad software). *Galleria mellonella* survival after *C. jejuni* infection were compared using unpaired *t*-test function.

## Figures and Tables

**Table 1:** CjDsbAs biochemical features.

Feature	CjDsbA1	CjDsbA2	EcDsbA	EcDsbL
**Catalytic motif**				
CXXC	CIHC	CTHC	CPHC	CPFC
cis-Pro	TcisP	TcisP	VcisP	VcisP
**Biochemical characteristics**				
Redox potential	−60 mV	−116 mV	−122 mV ^a^	−95 mV ^a^
Insulin reduction activity assay	**–**	**–**	**+ ^a^**	**– ^a^**
RNaseA oxidation activity assay	**+**	**++**	**++ ^a^**	**– ^a^**

^a^—from Grimshaw et al. [24].

**Table 2:** Pairwise structure and sequence comparison of *E. coli* and *C. jejuni* Dsb proteins. (In brackets are protein’s PDB IDs: 4TKY—DsbA from *E. coli* [41], 3C7M—DsbL from *E. coli* [24], 7PQ7—DsbA1 from *C. jejuni*, 7PQF—DsbA2 from *C. jejuni*).

	EcDsbA (4TKY)		EcDsbL (3C7M)		CjDsbA1 (7PQ7)		CjDsbA2 (7PQF)	
	Seq. ID (%)	RMSD [Å] (Aligned Residues)	Seq. ID (%)	RMSD (Å) (Aligned Residues)	Seq. ID (%)	RMSD (Å) (Aligned Residues)	Seq. ID (%)	RMSD (Å) (Aligned Residues)
**EcDsbA** **(4TKY)**	X	X	22.5	2.22 (173)	24.2	2.36 (131)	23.5	2.56 (136)
**EcDsbL** **(3C7M)**			X	X	28.3	1.78 (152)	39.8	1.26 (186)
**CjDsbA1** **(7PQ7)**					X	X	52.4	1.09 (187)
**CjDsbA2** **(7PQF)**							X	X

**Table 3:** Loops and catalytic motif sequences of *C. jejuni* and *E. coli* Dsb proteins.

	L1	L2	L3	CXXC
**EcDsbA**	^61^VNFMGG^66^	^147^LRGVPAM^153^	^162^NPQGMDTSNMDVFVQ^176^	^30^ CPHC^33^
**EcDsbL**	^58^LETKGE^63^	^158^AKIQGVPAY^166^	^175^YTKSIKSID^183^	^29^CPFC^32^
**CjDsbA1**	^57^VSLMNG^62^	^155^IAKTYGTPAF^164^	^173^NPSAINSMQ^181^	^28^CIHC^31^
**CjDsbA2**	^58^VSSMGD^63^	^157^ISQNYGTPAF^166^	^175^IPSAINSPE^183^	^29^CTHC^32^

**Table 4:** Proteins whose ratio was 0.55 or less in Δ*dsbA1*Δ*c8j_1298* comparing to *C. jejuni* 81116 wild-type strain (*p* < 0.05).

Locus Tag *C. jejuni* 81116	Gene	NCBI Protein ID	q-Value	Ratio Δ*dsbA1*Δ*c8j1298*/WT	Peptide Number	Description	^1^ SignalP Prediction	^2^ Cysteine Residues Number
**C8J_0082**		WP_002866718.1	NA	only in WT	4	Putative lipoprotein	Sec/SPII	4
**C8J_1462**	*pflA*	WP_002866855.1	NA	only in WT	2	Paralyzed flagellum protein	Sec/SPI	6
**C8J_0543**	*rppH (nudH)*	WP_002854797.1	NA	only in A1	2	RNA pyrophosphohydrolase		1
**C8J_0596**	*msrA*	WP_002866497.1	NA	only in WT	2	Peptide methionine sulfoxide reductase MsrA		4
**C8J_0412**	*mfrA*	WP_002867001.1	0.00010	0.30	54	Succinate dehydrogenase, flavoprotein subunit	Tat/SPI	12
**C8J_0241**		WP_002871921.1	0.00010	0.37	62	Trimethylamine-N-oxide reductase	Tat/SPI	10
**C8J_0413**	*mfrB*	WP_002854950.1	0.00010	0.23	45	Succinate dehydrogenase, iron–sulfur protein subunit		13
**C8J_1439**	*tupA*	WP_002877290.1	0.00010	0.27	29	Tungstate-binding protein TupA	Sec/SPI	2
**C8J_0558**	*peb4 (ppiC)*	WP_002876664.1	0.00010	0.38	32	Putative peptidyl-prolyl cis-trans isomerase Cbf2	Sec/SPI	0
**C8J_0731**	*napA*	WP_002866908.1	0.00010	0.52	91	Periplasmic nitrate reductase	Tat/SPI	12
**C8J_0040**	*cccC*	WP_002859743.1	0.00010	0.34	16	Putative cytochrome C	Sec/SPI	5
**C8J_0028**		WP_072238642.1	0.00017	0.35	27	Cytoplasmic L-asparaginase	Sec/SPII	0
**C8J_0079**	*aspA*	WP_002854440.1	0.00083	0.51	67	Aspartate ammonia-lyase		7
**C8J_1282**	*fumC*	WP_002877339.1	0.00296	0.51	55	Fumarate hydratase class II		5
**C8J_0955**	*livK*	WP_002853905.1	0.00715	0.25	18	Branched-chain amino-acid ABC transport system, periplasmic binding protein	Sec/SPI	3
**C8J_0335**		WP_012006644.1	0.01006	0.45	26	Cytochrome c551 peroxidase	Sec/SPI	4
**C8J_1099**	*cccA*	WP_002852762.1	0.02261	0.48	15	Cytochrome c553	Sec/SPI	3

^1^—type of predicted signal sequence evaluated by using SignalP; ^2^—number of cysteine residues present in immature protein sequence.

**Table 5:** Bacterial strains, plasmids and primers used in presented study (DBG Collection*—Department of Bacterial Genetics University of Warsaw Collection).

Name	Relevant Characteristics		Source/Ref.
***E. coli* STRAINS**			
TG1	*supE44 hsd*Δ *5 thi* Δ(*lac*^−^ *proAB*) F’ [*traD36 proAB^+^ lacI^q^ lacZ*Δ*M15*]		[75]
Rosetta(DE3)pLysS	F^−^ *ompT hsdS_B_*(r_B_^−^m_B_^−^) *gal dcm* (DE3) pLysS RARE (Cm^R^)		Novagen
BL21 (DE3)	F^−^ *ompT gal dcm lon hsdSB*(r_B_^−^ m_B_^−^) *λ*(DE3 [*lacI lacUV5-T7 gene 1 ind1 sam7 nin5*])		[76]
BL21C43 (DE3)	F^−^ *ompT gal dcm lon hsdSB*(r_B_^−^m_B_^−^) λ(DE3 [*lacI lacUV5-T7 gene 1 ind1 sam7 nin5*]) and two uncharacterized mutations		[77]
JCB816	MC1000 *phoR* λ102		[78]
JCB816/pMPM-A6	MC1000 *phoR* λ102 carrying pMPM-A6; Ap^R^		DBG Collection*
JCB817	MC1000 *phoR* λ102 *dsbA::kan1;* Km^R^		[78]
JCB817/pMPM-A6	MC1000 *phoR* λ102 *dsbA::kan1* carrying pMPM-A6; Km^R^ Ap^R^		DBG Collection*
JCB818	MC1000 *phoR* λ102 *dsbA::kan1, dsbB::kan;* Km^R^		[78]
JCB818/pMPM-A6	MC1000 *phoR* λ102 *dsbA::kan1, dsbB::Kan* carrying pMPM-A6; Km^R^ Ap^R^		DBG Collection*
BL21/*EcdsbA*	BL21 carrying *EcdsbA*^+^ in pET28a Km^R^		J.F. Collet Collection
KBO1436	Rosetta(DE3)pLysS carrying pUWM1430 (*CjdsbA1*^+^ in pET28a) Km^R^ Cm^R^		[36]
KBO1441	Rosetta(DE3)pLysS carrying pUWM1432 (*CjdsbA2*^+^ in pET24a) Km^R^ Cm^R^		[36]
C43(DE3)/pUWM1469	Rosetta(DE3)pLysS carrying pUWM1469 (*CjdsbB*^+^ in pET24a) Km^R^ Cm^R^		This study
AG1254	JCB817 carrying *ss’pelB-cjdsbA1*^+^ in pMPM-A6 Km^R^ Ap^R^		[32]
AG1256	JCB818 carrying *ss’pelB-cjdsbA1*^+^ in pMPM-A6 Km^R^ Ap^R^		[32]
AB1525	JCB817 carrying pUWM1523 (*ss’pelB- dsbA2*^+^ in pMPM-A6) Km^R^ Ap^R^		This study
AB1566	JCB818 carrying pUWM1523 (*ss’pelB- dsbA2*^+^ in pMPM-A6) Km^R^ Ap^R^		This study
*Campylobacter jejuni* 81116 STRAINS			
wild-type—81116 (NCTC11828)	Parental strain		[79]
KBO1	*CjdsbA1::cat*		[36]
KBO2	*CjdsbA1::aph3, dsbA2::cat*		[36]
AG2	*CjdsbA2::cat*		[32]
AG3	*CjdsbB::kan*		[32]
AG4	*CjdsbI::cat*		[32]
AB4	*CjdsbA1::cat, c8j_1298:aph3*		[36]
General cloning and expression vectors			
pJet 1.2 blunt	Ap^R^, CloneJET PCR Cloning Kit		Thermo Fisher Scientific
pET22b	Ap^R^; *ss’pelB*, IPTG inducible		Novagen
pET24a	Km^R^, IPTG inducible		Novagen
pMPM-A6	Ap^R^, Spec^R^, P_araBAD_		[37]
Plasmids for recombinant Dsb overexpression and purification			
pUWM1430	*CjdsbA1-His_6_* in pET28a		[36]
pUWM1432	*CjdsbA2-His_6_* in pET24a		[36]
pUWM1459	*CjdsbB* in pJet 1.2 blunt (to generate pUWM1469)		This study
pUWM1469	*CjdsbB-His_6_* in pET24a		This study
pET28a/*EcdsbA*	*EcdsbA* in pET28a		J.F. Collet Collection
Plasmid for complementation experiments			
pUWM1522	*CjdsbA2* in pET22b		This study
pUWM1523	*ss’pelB-cjdsbA2* in pMPM-A6		This study
Primers			
**Name**	**Sequence**	**Restriction Site**	**Source**
Primers for complementation			
c8j0811Ec_BamHI	GTCGGATCCGTTAAGTGAAGGTAAAG	BamHI	This study
c8j0811Ec_stopXhoI	GCACTCGAGAACTCATTTTTGTTTGCTAAG	XhoI	This study
Primers for recombinant proteins			
dsbB_pur_rev	AGTAAGCTTAACGACTTGATTTAAATGATTTAAACT	HindIII	This study
dsbB_pur_for	AGTCATATGAAAGATAATTGCAGAAAATTTTCACT	NdeI	This study

**Table 6:** Data collection and refinement statistics. Statistics for the highest-resolution shell are shown in parentheses.

	CjDsbA1		CjDsbA2
PDB ID	7PQ7	7PQ8	7PQF
Xray source	Elettra XRD2	BESSY 14.1	SLS PXIII
Wavelength (Å)	0.9789	0.9184	1.0
Resolution range (Å)	45.83–1.55 (1.64–1.55)	48.89–1.33 (1.41–1.33)	39.26–1.81 (1.92–1.81)
Space group	C2	P2_1_2_1_2_1_	P3_2_21
Unit cell (Å, °)	120.88 51.73 75.54 90.0 125.14 90.0	34.49 57.67 93.78 90.0 90.0 90.0	43.49 43.49 196.31 90.0 90.0 120.0
Total reflections	194386 (30,855)	307,623 (47,208)	283,766 (42,669)
Unique reflections	55,179 (8791)	81,341 (12,965)	20,471 (3072)
Multiplicity	3.52	3.78	13.86
Completeness (%)	98.6 (97.4)	97.8 (96.4)	99.2 (95.3)
Mean I/sigma(I)	16.31 (1.53)	8.94 (1.77)	14.60 (1.04)
Wilson B-factor	33.05	18.69	41.48
R-merge (%)	3.6 (69.3)	8.2 (67)	11.6 (271.4)
R-meas (%)	4.3 (81.6)	9.5 (78.3)	12.1 (281.7)
CC_1/2_	99.9 (77.2)	99.6 (65.5)	99.9 (63.2)
Reflections used in refinement	55,153 (5392)	42145	20,349 (1926)
R-work	0.1768 (0.3444)	0.1759	0.1990 (0.3609)
R-free	0.2009 (0.3550)	0.2192	0.2346 (0.3675)
Number of non-hydrogen atoms	3569	1942	1681
Macromolecules	3163	1631	1574
Solvent	383	311	107
Protein residues	379	191	194
RMS(bonds)	0.019	0.011	0.009
RMS(angles)	1.62	1.75	1.01
Ramachandran favored (%)	99.20	98.94	99.48
Ramachandran allowed (%)	0.80	1.06	0.52
Ramachandran outliers (%)	0	0	0
Rotamer outliers (%)	0.56	0.55	0.58
Clashscore	4.42	4.00	3.18
Average B-factor	37.96	14.02	41.93
Macromolecules	37.03	13.69	41.72
Solvent	44.59	27.04	45.01

## Data Availability

The X-ray crystal structures were deposited in PDB under accession codes 7PQ7, 7PQ8 and 7PQF. Diffraction images are available in the IRRMC repository (https://proteindiffraction.org), 7PQF at doi:10.18430/M3.IRRMC.6071; 7PQ7 at doi:10.18430/M3.IRRMC.6072; 7PQ8 at doi:10.18430/M3.IRRMC.6077. The mass spectrometry proteomics data were deposited to the ProteomeXchange Consortium via the PRIDE partner repository with the dataset identifier PXD029340 and 10.6019/PXD029340.

## Supplementary Materials

The following are available online at https://www.mdpi.com/article/10.3390/ijms222413451/s1.

## References

[1] Landeta C., Boyd D., Beckwith J. (2018). Disulfide bond formation in prokaryotes. Nat. Microbiol..

[2] Hatahet F., Boyd D., Beckwith J. (2014). Disulfide bond formation in prokaryotes: History, diversity and design. Biochim. Biophys. Acta.

[3] Manta B., Boyd D., Berkmen M. (2019). Disulfide bond formation in the periplasm of *Escherichia coli*. EcoSal Plus.

[4] Shouldice S.R., Heras B., Walden P.M., Totsika M., Schembri M.A., Martin J.L. (2011). Structure and function of DsbA, a key bacterial oxidative folding catalyst. Antioxid. Redox Signal..

[5] McMahon R.M., Premkumar L., Martin J.L. (2014). Four structural subclasses of the antivirulence drug target disulfide oxidoreductase DsbA provide a platform for design of subclass-specific inhibitors. Biochim. Biophys. Acta.

[6] Totsika M., Vagenas D., Paxman J.J., Wang G., Dhouib R., Sharma P., Martin J.L., Scanlon M.J., Heras B. (2018). Inhibition of Diverse DsbA Enzymes in Multi-DsbA Encoding Pathogens. Antioxid. Redox Signal..

[7] Yu J. (1998). Inactivation of DsbA, but not DsbC and DsbD, affects the intracellular survival and virulence of *Shigella flexneri*. Infect. Immun..

[8] Kurth F., Rimmer K., Premkumar L., Mohanty B., Duprez W., Halili M.A., Shouldice S.R., Heras B., Fairlie D.P., Scanlon M.J. (2013). Comparative sequence, structure and redox analyses of *Klebsiella pneumoniae* DsbA show that anti-virulence target DsbA enzymes fall into distinct classes. PLoS ONE.

[9] Kurth F., Duprez W., Premkumar L., Schembri M.A., Fairlie D.P., Martin J.L. (2014). Crystal structure of the dithiol oxidase DsbA enzyme from *Proteus mirabilis* bound non-covalently to an active site peptide ligand. J. Biol. Chem..

[10] Turcot I., Ponnampalam T.V., Bouwman C.W., Martin N.L. (2001). Isolation and characterization of a chromosomally encoded disulphide oxidoreductase from *Salmonella enterica* serovar Typhimurium. Can. J. Microbiol..

[11] Heras B., Totsika M., Jarrott R., Shouldice S.R., Guncar G., Achard M.E., Wells T.J., Argente M.P., McEwan A.G., Schembri M.A. (2010). Structural and functional characterization of three DsbA paralogues from *Salmonella enterica* serovar typhimurium. J. Biol. Chem..

[12] Banas A.M., Bocian-Ostrzycka K.M., Jagusztyn-Krynicka E.K. (2019). Engineering of the Dsb (disulfide bond) proteins—Contribution towards understanding their mechanism of action and their applications in biotechnology and medicine. Crit. Rev. Microbiol..

[13] McMahon R.M., Coincon M., Tay S., Heras B., Morton C.J., Scanlon M.J., Martin J.L. (2015). Sent packing: Protein engineering generates a new crystal form of *Pseudomonas aeruginosa* DsbA1 with increased catalytic surface accessibility. Acta Crystallogr. D Biol. Crystallogr..

[14] Lafaye C., Iwema T., Carpentier P., Jullian-Binard C., Kroll J.S., Collet J.F., Serre L. (2009). Biochemical and structural study of the homologues of the thiol-disulfide oxidoreductase DsbA in *Neisseria meningitidis*. J. Mol. Biol..

[15] Vivian J.P., Scoullar J., Robertson A.L., Bottomley S.P., Horne J., Chin Y., Wielens J., Thompson P.E., Velkov T., Piek S. (2008). Structural and biochemical characterization of the oxidoreductase NmDsbA3 from *Neisseria meningitidis*. J. Biol. Chem..

[16] Vivian J.P., Scoullar J., Rimmer K., Bushell S.R., Beddoe T., Wilce M.C., Byres E., Boyle T.P., Doak B., Simpson J.S. (2009). Structure and function of the oxidoreductase DsbA1 from *Neisseria meningitidis*. J. Mol. Biol..

[17] Ireland P.M., McMahon R.M., Marshall L.E., Halili M., Furlong E., Tay S., Martin J.L., Sarkar-Tyson M. (2014). Disarming *Burkholderia pseudomallei*: Structural and functional characterization of a disulfide oxidoreductase (DsbA) required for virulence in vivo. Antioxid. Redox Signal..

[18] Kurz M., Iturbe-Ormaetxe I., Jarrott R., Shouldice S.R., Wouters M.A., Frei P., Glockshuber R., O’Neill S.L., Heras B., Martin J.L. (2009). Structural and functional characterization of the oxidoreductase alpha-DsbA1 from *Wolbachia pipientis*. Antioxid. Redox Signal..

[19] Arts I.S., Ball G., Leverrier P., Garvis S., Nicolaes V., Vertommen D., Ize B., Tamu Dufe V., Messens J., Voulhoux R. (2013). Dissecting the machinery that introduces disulfide bonds in *Pseudomonas aeruginosa*. mBio.

[20] Christensen S., Groftehauge M.K., Byriel K., Huston W.M., Furlong E., Heras B., Martin J.L., McMahon R.M. (2016). Structural and biochemical characterization of *Chlamydia trachomatis* DsbA reveals a cysteine-rich and weakly oxidising oxidoreductase. PLoS ONE.

[21] Daniels R., Mellroth P., Bernsel A., Neiers F., Normark S., von Heijne G., Henriques-Normark B. (2010). Disulfide bond formation and cysteine exclusion in Gram-positive bacteria. J. Biol. Chem..

[22] Zhou Y., Cierpicki T., Jimenez R.H., Lukasik S.M., Ellena J.F., Cafiso D.S., Kadokura H., Beckwith J., Bushweller J.H. (2008). NMR solution structure of the integral membrane enzyme DsbB: Functional insights into DsbB-catalyzed disulfide bond formation. Mol. Cell.

[23] Inaba K., Murakami S., Suzuki M., Nakagawa A., Yamashita E., Okada K., Ito K. (2006). Crystal structure of the DsbB-DsbA complex reveals a mechanism of disulfide bond generation. Cell.

[24] Grimshaw J.P., Stirnimann C.U., Brozzo M.S., Malojcic G., Grutter M.G., Capitani G., Glockshuber R. (2008). DsbL and DsbI form a specific dithiol oxidase system for periplasmic arylsulfate sulfotransferase in uropathogenic *Escherichia coli*. J. Mol. Biol..

[25] Lin D., Kim B., Slauch J.M. (2009). DsbL and DsbI contribute to periplasmic disulfide bond formation in *Salmonella enterica* serovar Typhimurium. Microbiology.

[26] Totsika M., Heras B., Wurpel D.J., Schembri M.A. (2009). Characterization of two homologous disulfide bond systems involved in virulence factor biogenesis in uropathogenic *Escherichia coli* CFT073. J. Bacteriol..

[27] European Food Safety Authority, and European Centre for Disease Prevention and Control (2021). The European Union One Health 2019 Zoonoses Report. EFSA J..

[28] Burnham P.M., Hendrixson D.R. (2018). Campylobacter jejuni: Collective components promoting a successful enteric lifestyle. Nat. Rev. Microbiol..

[29] Tresse O., Alvarez-Ordonez A., Connerton I.F. (2017). Editorial: About the foodborne pathogen campylobacter. Front. Microbiol..

[30] Kaakoush N.O., Castano-Rodriguez N., Mitchell H.M., Man S.M. (2015). Global epidemiology of campylobacter infection. Clin. Microbiol. Rev..

[31] Bocian-Ostrzycka K.M., Grzeszczuk M.J., Dziewit L., Jagusztyn-Krynicka E.K. (2015). Diversity of the *Epsilonproteobacteria* Dsb (disulfide bond) systems. Front. Microbiol..

[32] Grabowska A.D., Wywial E., Dunin-Horkawicz S., Lasica A.M., Wosten M.M., Nagy-Staron A., Godlewska R., Bocian-Ostrzycka K., Pienkowska K., Laniewski P. (2014). Functional and bioinformatics analysis of two *Campylobacter jejuni* homologs of the thiol-disulfide oxidoreductase, DsbA. PLoS ONE.

[33] Roszczenko P., Radomska K.A., Wywial E., Collet J.F., Jagusztyn-Krynicka E.K. (2012). A novel insight into the oxidoreductase activity of *Helicobacter pylori* HP0231 protein. PLoS ONE.

[34] Denoncin K., Nicolaes V., Cho S.H., Leverrier P., Collet J.F. (2013). Protein disulfide bond formation in the periplasm: Determination of the in vivo redox state of cysteine residues. Methods Mol. Biol..

[35] Bocian-Ostrzycka K.M., Lasica A.M., Dunin-Horkawicz S., Grzeszczuk M.J., Drabik K., Dobosz A.M., Godlewska R., Nowak E., Collet J.F., Jagusztyn-Krynicka E.K. (2015). Functional and evolutionary analyses of *Helicobacter pylori* HP0231 (DsbK) protein with strong oxidative and chaperone activity characterized by a highly diverged dimerization domain. Front. Microbiol..

[36] Banas A.M., Bocian-Ostrzycka K.M., Plichta M., Dunin-Horkawicz S., Ludwiczak J., Placzkiewicz J., Jagusztyn-Krynicka E.K. (2020). C8J_1298, a bifunctional thiol oxidoreductase of *Campylobacter jejuni*, affects Dsb (disulfide bond) network functioning. PLoS ONE.

[37] Mayer M.P. (1995). A new set of useful cloning and expression vectors derived from pBlueScript. Gene.

[38] Dailey F.E., Berg H.C. (1993). Mutants in disulfide bond formation that disrupt flagellar assembly in *Escherichia coli*. Proc. Natl. Acad. Sci. USA.

[39] Wunderlich M., Otto A., Maskos K., Mucke M., Seckler R., Glockshuber R. (1995). Efficient catalysis of disulfide formation during protein folding with a single active-site cysteine. J. Mol. Biol..

[40] Ondo-Mbele E., Vives C., Kone A., Serre L. (2005). Intriguing conformation changes associated with the trans/cis isomerization of a prolyl residue in the active site of the DsbA C33A mutant. J. Mol. Biol..

[41] Duprez W., Premkumar L., Halili M.A., Lindahl F., Reid R.C., Fairlie D.P., Martin J.L. (2015). Peptide inhibitors of the *Escherichia coli* DsbA oxidative machinery essential for bacterial virulence. J. Med. Chem..

[42] Paxman J.J., Borg N.A., Horne J., Thompson P.E., Chin Y., Sharma P., Simpson J.S., Wielens J., Piek S., Kahler C.M. (2009). The structure of the bacterial oxidoreductase enzyme DsbA in complex with a peptide reveals a basis for substrate specificity in the catalytic cycle of DsbA enzymes. J. Biol. Chem..

[43] Grauschopf U., Winther J.R., Korber P., Zander T., Dallinger P., Bardwell J.C. (1995). Why is DsbA such an oxidizing disulfide catalyst?. Cell.

[44] Grzeszczuk M.J., Bocian-Ostrzycka K.M., Banas A.M., Roszczenko-Jasinska P., Malinowska A., Stralova H., Haas R., Meyer T.F., Jagusztyn-Krynicka E.K. (2018). Thioloxidoreductase HP0231 of *Helicobacter pylori* impacts HopQ-dependent CagA translocation. Int. J. Med. Microbiol..

[45] Hiniker A., Bardwell J.C. (2004). In vivo substrate specificity of periplasmic disulfide oxidoreductases. J. Biol. Chem..

[46] Andreesen J.R., Makdessi K. (2008). Tungsten, the surprisingly positively acting heavy metal element for prokaryotes. Ann. N. Y. Acad. Sci..

[47] Smart J.P., Cliff M.J., Kelly D.J. (2009). A role for tungsten in the biology of *Campylobacter jejuni*: Tungstate stimulates formate dehydrogenase activity and is transported via an ultra-high affinity ABC system distinct from the molybdate transporter. Mol. Microbiol..

[48] Rathbun K.M., Thompson S.A. (2009). Mutation of PEB4 alters the outer membrane protein profile of *Campylobacter jejuni*. FEMS Microbiol. Lett..

[49] Rathbun K.M., Hall J.E., Thompson S.A. (2009). Cj0596 is a periplasmic peptidyl prolyl cis-trans isomerase involved in *Campylobacter jejuni* motility, invasion, and colonization. BMC Microbiol..

[50] Atack J.M., Kelly D.J. (2009). Oxidative stress in *Campylobacter jejuni*: Responses, resistance and regulation. Future Microbiol..

[51] Boschi-Muller S., Branlant G. (2014). Methionine sulfoxide reductase: Chemistry, substrate binding, recycling process and oxidase activity. Bioorg. Chem..

[52] Oakland M., Jeon B., Sahin O., Shen Z., Zhang Q. (2011). Functional characterization of a lipoprotein-encoding operon in *Campylobacter jejuni*. PLoS ONE.

[53] Bleumink-Pluym N.M.C., Verschoor F., Gaastra W., van der Zeijst B.A.M., Fry B.N. (1999). A novel approach for the construction of a *Campylobacter* mutant library. Microbiology (Reading).

[54] Beeby M., Ribardo D.A., Brennan C.A., Ruby E.G., Jensen G.J., Hendrixson D.R. (2016). Diverse high-torque bacterial flagellar motors assemble wider stator rings using a conserved protein scaffold. Proc. Natl. Acad. Sci. USA.

[55] Gundogdu O., da Silva D.T., Mohammad B., Elmi A., Mills D.C., Wren B.W., Dorrell N. (2015). The *Campylobacter jejuni* MarR-like transcriptional regulators RrpA and RrpB both influence bacterial responses to oxidative and aerobic stresses. Front. Microbiol..

[56] Gundogdu O., da Silva D.T., Mohammad B., Elmi A., Wren B.W., van Vliet A.H., Dorrell N. (2016). The *Campylobacter jejuni* oxidative stress regulator RrpB is associated with a genomic hypervariable region and rltered oxidative stress resistance. Front. Microbiol..

[57] Price M.N., Dehal P.S., Arkin A.P. (2010). FastTree 2-approximately maximum-likelihood trees for large alignments. PLoS ONE.

[58] Tinsley C.R., Voulhoux R., Beretti J.L., Tommassen J., Nassif X. (2004). Three homologues, including two membrane-bound proteins, of the disulfide oxidoreductase DsbA in *Neisseria meningitidis*: Effects on bacterial growth and biogenesis of functional type IV pili. J. Biol. Chem..

[59] Taylor A.J., Kelly D.J. (2019). The function, biogenesis and regulation of the electron transport chains in *Campylobacter jejuni*: New insights into the bioenergetics of a major food-borne pathogen. Adv. Microb. Physiol..

[60] Howlett R.M., Hughes B.M., Hitchcock A., Kelly D.J. (2012). Hydrogenase activity in the foodborne pathogen *Campylobacter jejuni* depends upon a novel ABC-type nickel transporter (NikZYXWV) and is SlyD-independent. Microbiology (Reading).

[61] Weerakoon D.R., Borden N.J., Goodson C.M., Grimes J., Olson J.W. (2009). The role of respiratory donor enzymes in *Campylobacter jejuni* host colonization and physiology. Microb. Pathog..

[62] van der Stel A.X., van de Lest C.H.A., Huynh S., Parker C.T., van Putten J.P.M., Wosten M. (2018). Catabolite repression in *Campylobacter jejuni* correlates with intracellular succinate levels. Environ. Microbiol..

[63] Kassem I.I., Candelero-Rueda R.A., Esseili K.A., Rajashekara G. (2017). Formate simultaneously reduces oxidase activity and enhances respiration in *Campylobacter jejuni*. Sci. Rep..

[64] Sparacino-Watkins C., Stolz J.F., Basu P. (2014). Nitrate and periplasmic nitrate reductases. Chem. Soc. Rev..

[65] Rothery R.A., Workun G.J., Weiner J.H. (2008). The prokaryotic complex iron-sulfur molybdoenzyme family. Biochim. Biophys. Acta.

[66] Garg N., Taylor A.J., Kelly D.J. (2018). Bacterial periplasmic nitrate and trimethylamine-N-oxide respiration coupled to menaquinol-cytochrome c reductase (Qcr): Implications for electrogenic reduction of alternative electron acceptors. Sci. Rep..

[67] Mintmier B., McGarry J.M., Sparacino-Watkins C.E., Sallmen J., Fischer-Schrader K., Magalon A., McCormick J.R., Stolz J.F., Schwarz G., Bain D.J. (2018). Molecular cloning, expression and biochemical characterization of periplasmic nitrate reductase from *Campylobacter jejuni*. FEMS Microbiol. Lett..

[68] Imlay J.A. (2019). Where in the world do bacteria experience oxidative stress?. Environ. Microbiol..

[69] Liu Y.W., Kelly D.J. (2015). Cytochrome c biogenesis in *Campylobacter jejuni* requires cytochrome c6 (CccA; Cj1153) to maintain apocytochrome cysteine thiols in a reduced state for haem attachment. Mol. Microbiol..

[70] Champion O.L., Karlyshev A.V., Senior N.J., Woodward M., La Ragione R., Howard S.L., Wren B.W., Titball R.W. (2010). Insect infection model for *Campylobacter jejuni* reveals that O-methyl phosphoramidate has insecticidal activity. J. Infect. Dis..

[71] Senior N.J., Bagnall M.C., Champion O.L., Reynolds S.E., La Ragione R.M., Woodward M.J., Salguero F.J., Titball R.W. (2011). *Galleria mellonella* as an infection model for *Campylobacter jejuni* virulence. J. Med. Microbiol..

[72] Mehat J.W., Park S.F., van Vliet A.H.M., La Ragione R.M. (2018). CapC, a novel autotransporter and virulence factor of *Campylobacter jejuni*. Appl. Environ. Microbiol..

[73] Tang Y., Cawthraw S., Bagnall M.C., Gielbert A.J., Woodward M.J., Petrovska L. (2017). Identification of temperature regulated factors of *Campylobacter jejuni* and their potential roles in virulence. AIMS Microbiol..

[74] Hermans D., Van Deun K., Martel A., Van Immerseel F., Messens W., Heyndrickx M., Haesebrouck F., Pasmans F. (2011). Colonization factors of *Campylobacter jejuni* in the chicken gut. Vet. Res..

[75] Sambrook J., Russell D.W. (2001). Molecular Cloning: A Laboratory Manual.

[76] Studier F.W., Moffatt B.A. (1986). Use of bacteriophage T7 RNA polymerase to direct selective high-level expression of cloned genes. J. Mol. Biol..

[77] Miroux B., Walker J.E. (1996). Over-production of proteins in *Escherichia coli*: Mutant hosts that allow synthesis of some membrane proteins and globular proteins at high levels. J. Mol. Biol..

[78] Bardwell J.C., McGovern K., Beckwith J. (1991). Identification of a protein required for disulfide bond formation in vivo. Cell.

[79] Palmer S.R., Gully P.R., White J.M., Pearson A.D., Suckling W.G., Jones D.M., Rawes J.C., Penner J.L. (1983). Water-borne outbreak of *Campylobacter* gastroenteritis. Lancet.

[80] Studier F.W. (2005). Protein production by auto-induction in high density shaking cultures. Protein Expr. Purif..

[81] Wunderlich M., Glockshuber R. (1993). Redox properties of protein disulfide isomerase (DsbA) from *Escherichia coli*. Protein Sci..

[82] Roszczenko P., Grzeszczuk M., Kobierecka P., Wywial E., Urbanowicz P., Wincek P., Nowak E., Jagusztyn-Krynicka E.K. (2015). *Helicobacter pylori* HP0377, a member of the Dsb family, is an untypical multifunctional CcmG that cooperates with dimeric thioldisulfide oxidase HP0231. BMC Microbiol..

[83] AAT Bioquest I. Quest Calculate™ Protein Concentration Calculator. https://www.aatbio.com/tools/calculate-protein-concentration.

[84] Kabsch W. (2010). Xds. Acta Crystallogr. D Biol. Crystallogr..

[85] Krug M., Weiss M.S., Heinemann U., Mueller U. (2012). XDSAPP: A graphical user interface for the convenient processing of diffraction data using XDS. J. Appl. Crystallogr..

[86] Moriarty N.W., Grosse-Kunstleve R.W., Adams P.D. (2009). Electronic Ligand Builder and Optimization Workbench (eLBOW): A tool for ligand coordinate and restraint generation. Acta Crystallogr. D Biol. Crystallogr..

[87] Bunkoczi G., Echols N., McCoy A.J., Oeffner R.D., Adams P.D., Read R.J. (2013). Phaser.MRage: Automated molecular replacement. Acta Crystallogr. D Biol. Crystallogr..

[88] Terwilliger T.C., Grosse-Kunstleve R.W., Afonine P.V., Moriarty N.W., Zwart P.H., Hung L.W., Read R.J., Adams P.D. (2008). Iterative model building, structure refinement and density modification with the PHENIX AutoBuild wizard. Acta Crystallogr. D Biol. Crystallogr..

[89] McCoy A.J., Grosse-Kunstleve R.W., Adams P.D., Winn M.D., Storoni L.C., Read R.J. (2007). Phaser crystallographic software. J. Appl. Crystallogr..

[90] Emsley P., Lohkamp B., Scott W.G., Cowtan K. (2010). Features and development of Coot. Acta Crystallogr. D Biol. Crystallogr..

[91] Afonine P.V., Grosse-Kunstleve R.W., Echols N., Headd J.J., Moriarty N.W., Mustyakimov M., Terwilliger T.C., Urzhumtsev A., Zwart P.H., Adams P.D. (2012). Towards automated crystallographic structure refinement with phenix.refine. Acta Crystallogr. D Biol. Crystallogr..

[92] Murshudov G.N., Skubak P., Lebedev A.A., Pannu N.S., Steiner R.A., Nicholls R.A., Winn M.D., Long F., Vagin A.A. (2011). REFMAC5 for the refinement of macromolecular crystal structures. Acta Crystallogr. D Biol. Crystallogr..

[93] Malinowska A., Kistowski M., Bakun M., Rubel T., Tkaczyk M., Mierzejewska J., Dadlez M. (2012). Diffprot—Software for non-parametric statistical analysis of differential proteomics data. J. Proteomics.

[94] Elias J.E., Haas W., Faherty B.K., Gygi S.P. (2005). Comparative evaluation of mass spectrometry platforms used in large-scale proteomics investigations. Nat. Methods.

[95] proteom.pl MScan. http://proteom.ibb.waw.pl/mscan/.

[96] Bakun M., Karczmarski J., Poznanski J., Rubel T., Rozga M., Malinowska A., Sands D., Hennig E., Oledzki J., Ostrowski J. (2009). An integrated LC-ESI-MS platform for quantitation of serum peptide ladders. Application for colon carcinoma study. Proteomics Clin. Appl..

[97] Askoura M., Stintzi A. (2017). Using *Galleria mellonella* as an infection model for *Campylobacter jejuni* pathogenesis. Methods Mol. Biol..

[98] Ren G., Stephan D., Xu Z., Zheng Y., Tang D., Harrison R.S., Kurz M., Jarrott R., Shouldice S.R., Hiniker A. (2009). Properties of the thioredoxin fold superfamily are modulated by a single amino acid residue. J. Biol. Chem..

[99] Altschul S.F., Madden T.L., Schaffer A.A., Zhang J., Zhang Z., Miller W., Lipman D.J. (1997). Gapped BLAST and PSI-BLAST: A new generation of protein database search programs. Nucleic Acids Res..

[100] Armenteros J.J.A., Tsirigos K.D., Sonderby C.K., Petersen T.N., Winther O., Brunak S., von Heijne G., Nielsen H. (2019). SignalP 5.0 improves signal peptide predictions using deep neural networks. Nat. Biotechnol..

[101] Teufel F., Armenteros J.J.A., Johansen A.R., Gislason M.H., Pihl S.I., Tsirigos K.D., Winther O., Brunak S., Von Heijne G., Nielsen H. (2021). SignalP 6.0 achieves signal peptide prediction across all types using protein language models. bioRxiv.

[102] Baek M., DiMaio F., Anishchenko I., Dauparas J., Ovchinnikov S., Lee G.R., Wang J., Cong Q., Kinch L.N., Schaeffer R.D. (2021). Accurate prediction of protein structures and interactions using a three-track neural network. Science.

