# Supplementary material for: Interplay between DsbA1, DsbA2 and C8J_1298 Periplasmic Oxidoreductases of Campylobacter jejuni and Their Impact on Bacterial Physiology and Pathogenesis
Source: Int J Mol Sci. 2021 Dec 15;22(24):13451. doi: 10.3390/ijms222413451 (PMC8708908; doi:10.3390/ijms222413451)
Supplement: Supplementary file 1 [file ijms-22-13451-s001.zip › ijms-1461809-supplementary/Figure S5.pdf]

(A)

```
LivJ_C.jejuni    1  MKKLTLTLSVLTM----VNCLYAKDINIGVVLPLTGTVAAYGQDVFNGIELANKLQPKLS--NGDVIKLI   64
LivK_C.jejuni    1  MKKSLILASILSL------SLSAAEVKIGVVLPLSGATAAYGQSALEGIKLANSMQSALS--NGDKVSLA   62
LivJ_E.coli      1  MNIKGKALLAGCIALAFSNMALAEDIKVAVVGAMSGPVAQYGDQEFTGAEQAVADINAKGGIKGNKLQIV   70
LivK_E.coli      1  MKRNAKTIIAGMIALAISHTAMADDIKVAVVGAMSGPIAQWGDMEFNGARQAIKDINAKGGIKGDKLVGV   70
Consensus_ss:        hhhhhhhhhhhhhhh         eeeeeee      hhhhhhhhhhhhhhhhhhhhh     eeeeee

LivJ_C.jejuni   65  TIDTKGDKLETSNGVNRLIATDKVLGIIGEATTPNTIQAISIAEEKKIPLIAPVASGDKLLDK-KKYASR  133
LivK_C.jejuni   63  IIDTKGDKLESSSGANRLVSQDKVIGLIGEMVTANTLQVMRVAEDNKIPLIAPAATGDRLLDK-KIYSSR  131
LivJ_E.coli     71  KYDDACDPKQAVAVANKVVND-GIKYVIGHLCSSSTQPASDIYEDEGILMITPAATAPELTARGYQLILR  139
LivK_E.coli     71  EYDDACDPKQAVAVANKIVND-GIKYVIGHLCSSSTQPASDIYEDEGILMISPGATNPELTQRGYQHIMR  139
Consensus_ss:       eee    hhhhhhhhhhhhh     eeee   hhhhhhhhhhhhh   eeeee    hhh      eeee

LivJ_C.jejuni  134  VCFKDSFQGDKFATYVSKDLGLKNAVIIIDQSNVYSLGLARAFENSFKNNGGKIIKKLVINSGDKDFRAV  203
LivK_C.jejuni  132  VCFMDSFQGSSLAKYVFSKLNYKSAVIVVDQSTDYSLGLAKAFEKQYKSNGGQILRILRVNSGDKDFRAI  201
LivJ_E.coli    140  TTGLDSDQGPTAAKYILEKVKPQ-RIAIVHDKQQYGEGLARAVQDGLKKGNANVVFFDGITAGEKDFSTL  208
LivK_E.coli    140  TAGLDSSQGPTAAKYILETVKPQ-RIAIIHDKQQYGEGLARSVQDGLKAANANVVFFDGITAGEKDFSAL  208
Consensus_ss:       e   hhhhhhhhhhhhhhh    eeeeeee   hhhhhhhhhhhhhhhh   eeeeeeee      hhhh

LivJ_C.jejuni  204  VSQLKSLNPDFVYMPIYHPEAALIARQARQIGFDKLLVAGDGVNNQTFIDLGGSAVNGVIFTDSFDYNSP  273
LivK_C.jejuni  202  VAQVKSLNPEFIFLPLYYSEASLFARQSKLAGLNIPMGSADGVADQTFISLAGDASEGYIFTDSFDANNP  271
LivJ_E.coli    209  VARLKKENIDFVYYGGYHPEMGQILRQARAAGLKTQFMGPEGVANVSLSNIAGESAEGLLVTKPKNY--D  276
LivK_E.coli    209  IARLKKENIDFVYYGGYYPEMGQMLRQARSVGLKTQFMGPEGVGNASLSNIAGDAAEGMLVTMPKRY--D  276
Consensus_ss:       hhhhhh    eeeee   hhhhhhhhhhhh     eeeeee    hhhhhhhhhhh  eeeeee

LivJ_C.jejuni  274  STQLGKDFVAAYEKVKGTKELPAFSAMGADAYFVMLNAMNACVDNLSSECINSKIHQTKDFQAVGGVISI  343
LivK_C.jejuni  272  TTKLSKEFISVYEKAKGTKEVPNFSAMGADAYFVMLNAMNACVENLTSKCVNEKIHQTKNYQGVSGVISI  341
LivJ_E.coli    277  QVPANKPIVDAIKAKKQD-PSGAFVWTTYAALQSLQAGLNQS---DDPAEIAKYLKAN-SVDTVMGPLTW  341
LivK_E.coli    277  QDPANQGIVDALKADKKD-PSGPYVWITYAAVQSLATALERTGS-DEPLALVKDLKAN-GANTVIGPLNW  343
Consensus_ss:         hhhhhhhhhhhhhh      hhhhhhhhhhhhhhhhhhhh     hhhhhhhhh         eeeee

LivJ_C.jejuni  344  DESGNAIR-SVVIKEIQNQKQNYKTIINP  371
LivK_C.jejuni  342  DQTGNATR-SVVVKEIKNQKQNYKDIINP  369
LivJ_E.coli    342  DEKGDLKGFEFGVFDWHANGTATDAK---  367
LivK_E.coli    344  DEKGDLKGFDFGVFQWHADGSSTAAK---  369
Consensus_ss:                 eeeeeee  eeeeeeee
```

(B)

	EcLivJ	EcLivK	CjLivJ	CjLivK
**EcLivJ**	-	(100% QC) **77%**	(92% QC) **28%**	(70% QC) **30%**
**EcLivK**	(100% QC) **77%**	-	(75% QC) **29%**	(66% QC) **28%**
**CjLivJ**	(93% QC) **28%**	(74% QC) **29%**	-	(94% QC) **64%**
**CjLivK**	(73% QC) **29%**	(69% QC) **28%**	(100% QC) **62%**	-
