# Supplementary material for: Interplay between DsbA1, DsbA2 and C8J_1298 Periplasmic Oxidoreductases of Campylobacter jejuni and Their Impact on Bacterial Physiology and Pathogenesis
Source: Int J Mol Sci. 2021 Dec 15;22(24):13451. doi: 10.3390/ijms222413451 (PMC8708908; doi:10.3390/ijms222413451)
Supplement: Supplementary file 1 [file ijms-22-13451-s001.zip › ijms-1461809-supplementary/Supplementary Figures description.pdf]

**Supplementary Figure S1.** The redox equilibrium CjDsbA1 with glutathione is twice as high as CjDsbA2, the latest corresponds to that determined for EcDsbA. The fraction of reduced (R) CjDsbA1/CjDsbA2 were determined in various glutathione (GSH)/glutathione disulfide (GSSG) ratios using AMS reagent. Fractions (band intensity) of reduced CjDsbA1/CjDsbA2 were determined using Image-Lab (BIO-RAD) after resolving on 16% SDS-PAGE. The standard redox potential was calculated from the Nernst equation using the glutathione standard potential. (A) The bars represent the average of three independent experiments, with two technical repetitions (n = 3). (B) The result of one representative experiment is presented.

**Supplementary Figure S2.** No cross-reactivity is observed between anti-CjDsbA1 and anti-CjDsbA2 serum. Proteins (whole cell lysates) from *C. jejuni* 81116 WT, Δ*cjdsbA1*, Δ*cjdsbA2* strains were separated by 12% SDS-PAGE, then Western blot analysis with rabbit anti-CjDsbA1 (A) or anti-CjDsbA2 (B) serum were performed. * - unspecific serum reaction

**Supplementary Figure S3.** CjDsbA1 *in vivo* redox state depends on CjDsbB presence in *C.jejuni* cells. Bacterial cell cultures were treated with 10% TCA (trichloroacetic acid) and AMS (4-acetamido-4′-maleimidylstilbene-2,2′-disulfonic acid). The controls were reduced (red; treated with DTT and AMS) and oxidized (ox; untreated with AMS) wild type cellular proteins. Samples were electrophoretically separated under non-reducing conditions using 16% polyacrylamide gel with sucrose addition. Then, Western blot analysis using specific anti-CjDbA1 serum was performed.

**Supplementary Figure S4.** *C. jejuni dsbA1* (A) and *dsbA2* (B) were cloned into pMPM-A6 under inducible arabinose promoter and expressed in *E. coli* cells. *E. coli* strains carrying recombinant plasmids were cultured with (+) or without (-) 0.2% arabinose (Ara). Proteins (whole cell lysates) from analyzed strains were separated by 12% SDS-PAGE, electrotransferred onto a nitrocellulose membrane and developed with rabbit anti-CjDsbA1 (A) or anti-CjDsbA2 (B) serum.

**Supplementary Figure S5.** (A) Structure-based, multiple sequence alignment of LivJ and LivK proteins from *E. coli* and *C. jejuni* obtained with PROMALS3D. The conserved, bridge-forming cysteines are indicated with a yellow background. The red and blue highlights indicate secondary structure elements (helices and beta strands, respectively). (B) The relative similarity of *E. coli* and *C. jejuni* Liv proteins. QC denotes query coverage.
