# Supplementary material for: Interplay between DsbA1, DsbA2 and C8J_1298 Periplasmic Oxidoreductases of Campylobacter jejuni and Their Impact on Bacterial Physiology and Pathogenesis
Source: Int J Mol Sci. 2021 Dec 15;22(24):13451. doi: 10.3390/ijms222413451 (PMC8708908; doi:10.3390/ijms222413451)
Supplement: Supplementary file 1 [file ijms-22-13451-s001.zip › ijms-1461809-supplementary/Supplementary Table S1.pdf]

Table S1. Proteins identified by MS analysis. Periplasmic proteome of *C. jejuni* 81116 wt strain and double mutated strain Δ*cjdsbA1*Δc8j_1298 was compared. Table presents only those proteins which ratio in Δ*cjdsbA1*Δ*c8j_1298* strain to wt strain was 0.55 or less.
(*Uniprot ID/locustag in most cases is determined for *C. jejuni* NCTC 11168, when protein was identified only for *C. jejuni* 81-176 strain we listed identifiers comming from that strain; NI** – homolog protein not identified in *C. jejuni* 81116 strain

**Uniprot ID***	**Locustag *C. jejuni* 11168 or *C. jejuni* 81-176 ***	**locustag *C. jejuni* 81116**	**NCBI protein ID** (for *C. jejuni* 81116)	**q-value**	**Ratio ΔdsbA1Δc8j1298 /WT**	**peptide number**	**description**	**SignalP prediction**
**Q0PC48**	Cj0089	C8J_0082	WP_002866718.1	NA	only in WT	4	Putative lipoprotein	Sec/SPII
**Q0P863**	Cj1565c	C8J_1462	WP_002866855.1	NA	only in WT	2	Paralysed flagellum protein	Sec/SPI
**Q9PHT5**	Cj0581	C8J_0543	WP_002854797.1	NA	only in A1	2	RNA pyrophosphohydrolase	
**Q9PHN0**	Cj0637c	C8J_0596	WP_002866497.1	NA	only in WT	2	Peptide methionine sulfoxide reductase MsrA	
**Q0PB73**	Cj0437	C8J_0412	WP_002867001.1	0.00010	0.30	54	Succinate dehydrogenase, flavoprotein subunit	Tat/SPI
**Q0PBP1**	Cj0264c	C8J_0241	WP_002871921.1	0.00010	0.37	62	Trimethylamine-N-oxide reductase	Tat/SPI
**Q0PB72**	Cj0438	C8J_0413	WP_002854950.1	0.00010	0.23	45	Succinate dehydrogenase, iron-sulfur protein subunit	
**Q0P885**	Cj1540	C8J_1439	WP_002877290.1	0.00010	0.27	29	Tungstate-binding protein TupA	Sec/SPI
**Q0PAS1**	Cj0596	C8J_0558	WP_002876664.1	0.00010	0.38	32	Putative peptidyl-prolyl cis-trans isomerase Cbf2	Sec/SPI
**Q9PPD9**	Cj0780	C8J_0731	WP_002866908.1	0.00010	0.52	91	Periplasmic nitrate reductase	Tat/SPI
**Q0PC89**	Cj0037c	C8J_0040	WP_002859743.1	0.00010	0.34	16	Putative cytochrome C	Sec/SPI
**Q0PC96**	Cj0029	C8J_0028	WP_072238642.1	0.00017	0.35	27	Cytoplasmic L-asparaginase	Sec/SPII
**Q0P8E1**	Cj1476c	C8J_1381	WP_002877436.1	0.00023	0.69	81	Pyruvate-flavodoxin oxidoreductase	
**Q0PC50**	Cj0087	C8J_0079	WP_002854440.1	0.00083	0.51	67	Aspartate ammonia-lyase	
**O69294**	Cj1364c	C8J_1282	WP_002877339.1	0.00296	0.51	55	Fumarate hydratase class II	
**Q0P9N4**	Cj1018c	C8J_0955	WP_002853905.1	0.00715	0.25	18	Branched-chain amino-acid ABC transport system, periplasmic binding protein	Sec/SPI
**Q0PBF1**	Cj0358	C8J_0335	WP_012006644.1	0.01006	0.45	26	Cytochrome c551 peroxidase	Sec/SPI
**Q0P9A3**	Cj1153	C8J_1099	WP_002852762.1	0.02261	0.48	15	Cytochrome c553	Sec/SPI
**A0A0H3PHN8**	CJJ81176_0836	C8J_0766	WP_002866967.1	0.06950	0.45	17	Amino acid-binding protein	Sec/SPI
**Q0PBP0**	Cj0265c	C8J_0242	WP_009881977.1	0.11800	0.36	7	Putative cytochrome C-type haem-binding periplasmic protein	Sec/SPI
**A0A0H3P9I6**	CJJ81176_0884	C8J_0815	WP_002865930.1	0.12942	0.32	8	Cytochrome c family protein, degenerate	Sec/SPI
**Q0PBB6**	Cj0394c	C8J_0369	WP_002854234.1	0.13552	0.52	4	Type III pantothenate kinase	
**Q0P987**	Cj1169c	C8J_1113	WP_002822505.1	0.14064	0.07	6	Putative periplasmic protein	Sec/SPII
**Q0P8G9**	Cj1445c	C8J_1352	WP_002851300.1	0.16531	0.34	5	Capsule polysaccharide export system inner membrane protein	
**Q9PIE2**	Cj0360	C8J_0336	WP_002866639.1	0.22813	0.53	9	Phosphoglucosamine mutase	
**P0C633**	Cj1029c	C8J_0966	WP_002852941.1	0.27848	0.38	5	Outer membrane liproprotein MapA	Sec/SPII
**A0A0H3P9J4**	CJJ81176_0882	C8J_0813	WP_002865932.1	0.27983	0.48	13	Arylsulfate sulfotransferase, degenerate	Sec/SPI
**Q0P8K8**	Cj1406c	C8J_1320	WP_002932559.1	0.28483	0.29	5	Putative periplasmic protein	Sec/SPI

**Q0P819**	Cj1609	C8J_1510	WP_002866588.1	0.36017	0.37	7	Putative sulfate adenylyltransferase	
**Q0P9Z0**	Cj0909	C8J_0845	WP_002856967.1	0.36997	0.30	11	Putative periplasmic protein	Sec/SPI
**Q0PBK6**	Cj0303c	C8J_0280	WP_002857589.1	0.37929	0.35	10	Putative molybdate-binding lipoprotein	Sec/SPII
**A0A0H3PDT4**	CJJ81176_1617	C8J_1528	WP_002856602.1	0.38723	0.15	4	PepSY_like domain-containing protein	Sec/SPI
**A0A0H3PA35**	CJJ81176_0880	C8J_0811	WP_002865934.1	0.39565	0.44	8	Thiol:disulfide interchange protein DsbA	Sec/SPI
**A0A0H3PA64**	CJJ81176_0067	C8J_0033	WP_012006617.1	0.40641	0.55	36	Gamma-glutamyltransferase	Sec/SPI
**Q0P966**	Cj1190c	C8J_1134	WP_002877106.1	0.51006	0.09	3	Bipartate energy taxis response protein cetA	
**Q0PA57**	Cj0833c	C8J_0780	WP_002866956.1	0.51304	0.44	5	Putative oxidoreductase	
**Q0P9N3**	Cj1019c	C8J_0956	WP_002866056.1	0.51583	0.43	18	Branched-chain amino-acid ABC transport system periplasmic binding protein	Sec/SPI
**Q0P8L8**	Cj1394	C8J_1309	WP_002877369.1	0.52206	0.48	16	Adenylosuccinate lyase	
**Q0PAQ4**	Cj0613	C8J_0575	WP_002825033.1	0.53194	0.27	11	Putative periplasmic phosphate binding protein	
**Q9PID1**	Cj0371	C8J_0347	WP_002854351.1	0.54071	0.36	7	UPF0323 lipoprotein Cj0371	Sec/SPII
**A0A0H3PA46**	CJJ81176_1678	C8J_1583	WP_002877023.1	0.55804	0.18	7	DNA topoisomerase 1	
**Q0P8U3**	Cj1314c	C8J_1248	WP_002866282.1	0.55922	0.49	5	Putative imidazole glycerol phosphate synthase subunit hisF2	
**Q0PC19**	Cj0118	C8J_0111	WP_002866734.1	0.55959	0.23	7	ATP_bind_3 domain-containing protein	
**Q0PBD3**	Cj0377	C8J_0353	WP_002857315.1	0.57612	0.52	2	Putative AAA family ATPase	
**Q0P9Z2**	Cj0906c	C8J_0843	WP_002865910.1	0.57689	0.53	3	Putative periplasmic protein	Sec/SPI
**A1W0G5**	CJJ81176_1196	C8J_1125	WP_002866193.1	0.62675	0.54	3	Elongation factor Ts	
**Q0PAI0**	Cj0699c	C8J_0666	WP_002854995.1	0.62995	0.55	17	Glutamine synthetase	
**Q9PJ53**	Cj0066c	C8J_0059	WP_002852001.1	0.63571	0.49	7	3-dehydroquinate dehydratase	
**Q0P8A3**	Cj1516	C8J_1419	WP_002867060.1	0.63661	0.38	3	Putative periplasmic oxidoreductase	Tat/SPI
**Q9PNC3**	Cj1172c	C8J_1116	WP_002866185.1	0.64862	0.45	6	Probable transcriptional regulatory protein Cj1172c	
**Q0PBX4**	Cj0163c	C8J_0160	WP_002854663.1	0.64862	0.45	3	Uncharacterized protein	
**Q9PHM1**	Cj0646	C8J_0605	WP_002852227.1	0.65354	0.51	3	Endolytic peptidoglycan transglycosylase RlpA	Sec/SPII
**Q0PBA0**	Cj0410	C8J_0385	WP_002854302.1	0.65423	0.50	6	Fumarate reductase iron-sulfur subunit	
**Q0P9X8**	Cj0921c	C8J_0858	WP_002865903.1	0.66635	0.33	48	Major cell-binding factor	Sec/SPI
**Q9PIS2**	Cj0207	C8J_0196	WP_002851726.1	0.66635	0.39	5	Translation initiation factor IF-3	
**Q0PA47**	Cj0843c	C8J_0790	WP_002861483.1	0.67304	0.55	8	Putative secreted transglycosylase	Sec/SPI
**Q0PBJ3**	Cj0316	C8J_0293	WP_002866665.1	0.68210	0.21	3	Bifunctional chorismate mutase/prephenate dehydratase	
**Q0PB54**	Cj0459c	C8J_0432	WP_002851103.1	0.69391	0.54	5	HP0268 domain-containing protein	
**Q0P7V9**	Cj1670c	C8J_1571	WP_002866836.1	0.69774	0.38	5	Putative periplasmic protein	Sec/SPI
**A0A0H3PAA5**	CJJ81176_0419	C8J_0371	WP_002865885.1	0.71779	0.49	3	Lipoprotein, putative	Sec/SPII
**A0A0H3PAM0**	CJJ81176_0310	C8J_0260	WP_002857311.1	0.72592	0.55	4	Histidine kinase	
**Q0PA24**	Cj0872	C8J_0814	WP_002865931.1	0.74268	0.34	5	Thiol:disulfide interchange protein DsbA	Sec/SPI
**Q0P7X0**	Cj1659	C8J_1561	WP_002778842.1	0.74296	0.49	12	Periplasmic protein p19	Sec/SPI
**Q0P8J8**	Cj1416c	C8J_0637	WP_002877674.1	0.76348	0.40	5	Putative sugar-1-phosphate nucleotidyltransferase CTP:phosphoglutamine cytidylyltransferase	

**Q0PC72**	Cj0061c	C8J_0054	WP_002851943.1	0.76548	0.48	5	RNA polymerase sigma factor for flagellar operon	
**Q0PAR2**	Cj0605	C8J_0567	WP_002866476.1	0.78667	0.43	5	Putative amidohydrolase	
**Q0P9E8**	Cj1106	C8J_1047	WP_002856169.1	0.82742	0.53	2	Putative periplasmic thioredoxin	Sec/SPII
**Q9PI18**	Cj0491	C8J_0453	WP_002866973.1	0.85245	0.51	3	30S ribosomal protein S12	
**Q9PNH7**	Cj1117c	C8J_1058	WP_002866133.1	0.88173	0.53	2	Ribosomal protein L11 methyltransferase	
**Q0P8F0**	Cj1465	C8J_1371	WP_002826016.1	0.88637	0.37	4	Uncharacterized protein	
**Q0PBD4**	Cj0376	C8J_0352	WP_002868686.1	0.90631	0.47	3	Putative periplasmic protein	Sec/SPI
**Q9PMK5**	Cj1455	C8J_1361	WP_002867022.1	0.93469	0.45	8	Peptide chain release factor 2	
**A0A0H3PHM5**	CJJ81176_0858	C8J_0788	WP_002856787.1	0.94089	0.43	5	Molybdopterin-guanine dinucleotide biosynthesis protein MobB	
**Q0P9G5**	Cj1089c	C8J_1030	WP_002858150.1	0.95413	0.43	3	Uncharacterized protein	
**Q9PLX0**	Cj1708c	C8J_1612	WP_002779353.1	0.96946	0.47	5	30S ribosomal protein S10	
**Q0PAX0**	Cj0546	C8J_0507	WP_002866428.1	0.97938	0.50	5	3-octaprenyl-4-hydroxybenzoate carboxy-lyase, putative	
**Q0P8N7**	Cj1372	C8J_1291	WP_002866711.1	0.98089	0.52	5	Toluene tolerance protein, putative	Sec/SPI
**Q0PBS4**	Cj0230c	C8J_0208	WP_012006633.1	0.98623	0.44	10	Nicotinate phosphoribosyltransferase	
**Q0P9V7**	Cj0942c	C8J_0885	WP_002866879.1	1.00000	0.45	2	Protein translocase subunit SecA	
**Q0PCC0**	Cj0005c	C8J_0005	WP_002866803.1	1.00000	0.45	10	Molydopterin containing oxidoreductase	Tat/SPI
**Q0P8R5**	Cj1344c	C8J_1260	WP_002892992.1	1.00000	0.32	3	tRNA N6-adenosine threonylcarbamoyltransferase	
**A0A0H3P9J5**	CJJ81176_0921	C8J_0850	WP_002865905.1	1.00000	0.31	3	Invasion antigen B	
