# Supplementary figures and images for: Interplay between DsbA1, DsbA2 and C8J_1298 Periplasmic Oxidoreductases of Campylobacter jejuni and Their Impact on Bacterial Physiology and Pathogenesis
Source: Int J Mol Sci. 2021 Dec 15;22(24):13451. doi: 10.3390/ijms222413451 (PMC8708908; doi:10.3390/ijms222413451)

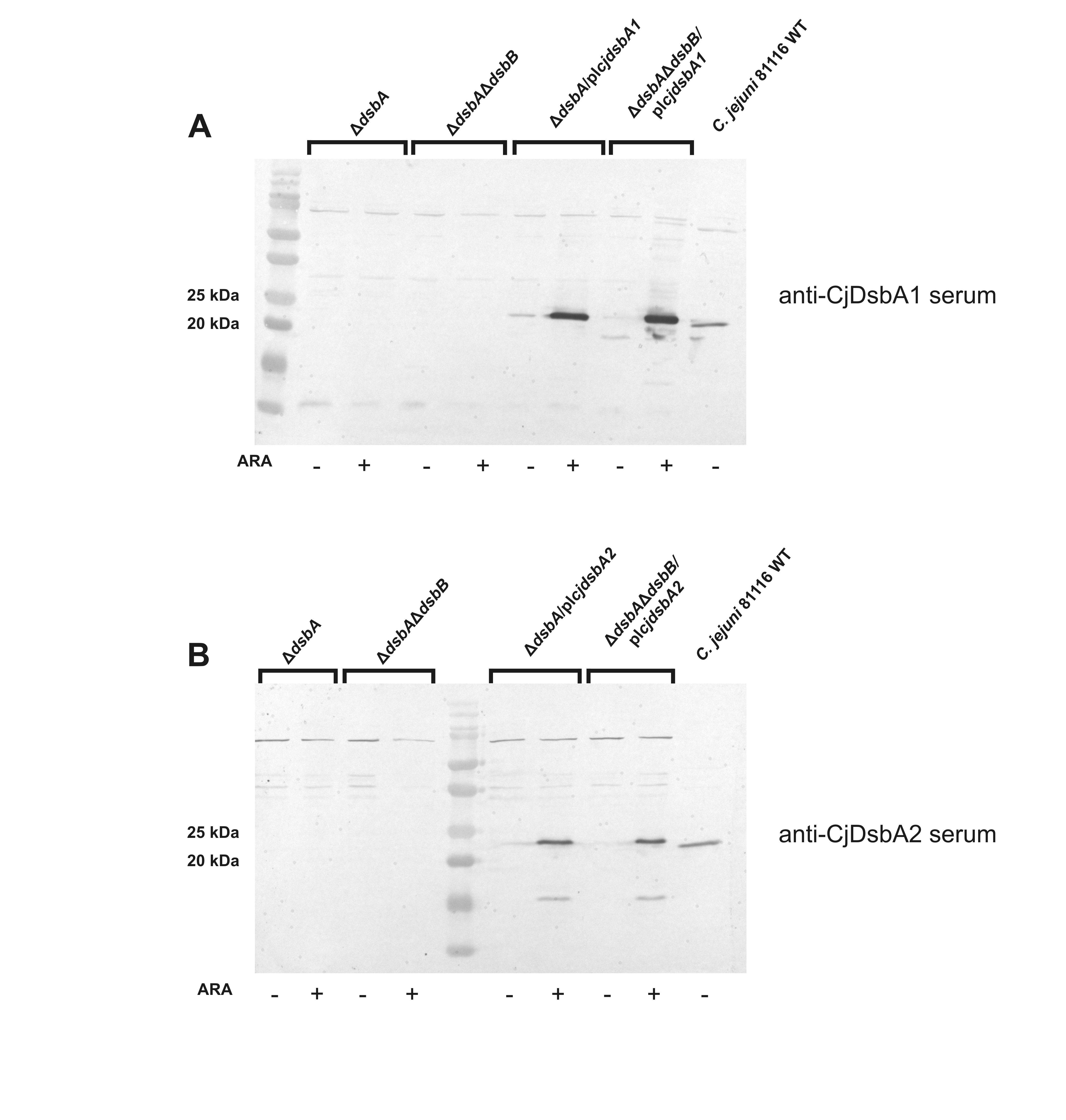

Supplement: Supplementary file 1 [file ijms-22-13451-s001.zip › ijms-1461809-supplementary/Figure S4.tif]
